# Engineered extracellular vesicles demonstrate altered endocytosis and biodistribution and have superior oral siRNA delivery efficiency compared to lipid nanoparticles

**DOI:** 10.1016/j.ijpx.2025.100428

**Published:** 2025-10-26

**Authors:** Ning Ding, Armond Daci, Vanesa Krasniqi, Rachel Butler, Alan Goddard, Qing Guo, Yunyue Zhang, Jizhou Zhong, K.L. Andrew Chan, Maya Thanou, Driton Vllasaliu

**Affiliations:** a Institute of Pharmaceutical Science, School of Cancer and Pharmaceutical Science, King's College London, London SE1 9NH, United Kingdom; b Department of Pharmacy, Faculty of Medicine, University of Prishtina, Prishtina 10000, Kosovo; c Aston Institute for Membrane Excellence and School of Biosciences, College of Health and Life Sciences, Aston University, Birmingham, UK

**Keywords:** Extracellular vesicles (EVs), RNA delivery, siRNA delivery, Oral RNA therapy, EV-LNP hybrids

## Abstract

Oral administration of RNA therapeutics remains a major unsolved challenge due to currently insurmountable biological barriers. Extracellular vesicles (EVs) are natural carriers capable of traversing the intestinal barrier, but inefficient RNA loading into EVs in general severely limits the application of EVs for RNA delivery. Here, we utilize a microfluidic engineering platform to generate milk-derived EV-lipid nanoparticle (EV-LNP) hybrids for oral delivery of RNA. The process produced uniform nanoparticles (133 nm, polydispersity index 0.19) with >45 % dual-positive fusion efficiency, significantly outperforming freeze–thaw hybridization. Compared to conventional LNPs, EV-LNP hybrids exhibited lower cytotoxicity, altered epithelial uptake pathways, and markedly improved intestinal epithelial transport. Importantly, the hybrids retained gene-silencing efficacy following exposure to simulated intestinal fluids, achieving 40–60 % glyceraldehyde 3-phosphate dehydrogenase knockdown in Caco-2 cells, which was superior to LNPs. Oral gavage in mice revealed preferential colonic accumulation of EV-LNP hybrids compared to native EVs or LNPs, indicating strong potential for local RNA therapy in gut diseases such as colitis. Collectively, this study establishes a scalable, bioinspired delivery platform that addresses key translational barriers for oral RNA therapeutics and enables targeted delivery to the colon.

## Graphical abstract

**Figure:**
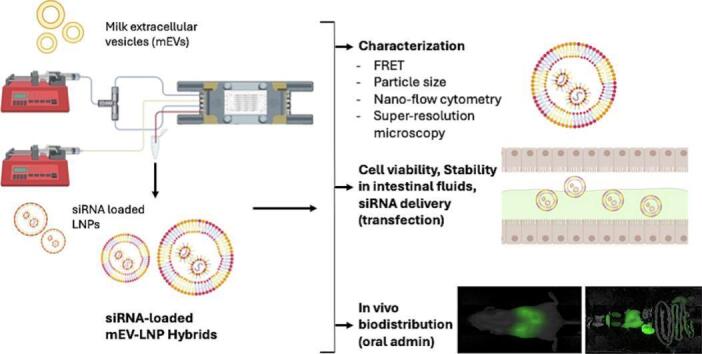

## 1 Introduction

RNA therapies, including siRNA and mRNA, have shown transformative clinical impact. Lipid nanoparticles (LNPs), the current state of art in RNA (mRNA and siRNA) delivery and key component of mRNA COVID-19 vaccines and other clinically approved RNA-based therapies, have had a profound impact on the clinical success of these therapies. However, the drawbacks of current LNP systems are costs, immune-related toxicity (Lee et al., 2023) and acute side effects (Moghimi and Simberg, 2022), while milk-derived extracellular vesicles (mEVs) are naturally stable in gastrointestinal (GI) fluids and capable of crossing intestinal barriers.(Ball et al., 2018).

Extracellular vesicles (EVs) are nature's cargo delivery systems, designed to transfer RNA molecules between cells. EVs show a remarkable ability to cross biological barriers, which is unmatched by synthetic delivery systems. EVs have been found to outperform synthetic RNA nanocarriers, delivering RNA several orders of magnitude more efficiently than LNPs (Murphy et al., 2021). EVs may offer a safer and more effective delivery system alternative to LNPs since they are non-immunogenic (Herrmann et al., 2021). However, the key barrier that stands in the way of utilizing EVs for delivery of RNA therapies, including via oral administration, is the difficulty of loading macromolecular drug payloads.

Several approaches for loading macromolecules into EVs have been reported, but most exogenous loading approaches are damaging, producing undesirable effects, such as inactivation of the drug or EV components (e.g. surface proteins), EV aggregation, and compromised tissue-penetrating properties of native EVs (Fu et al., 2020). Current EV drug loading strategies are highly inefficient for RNA (Aslan et al., 2021; Sutaria et al., 2017), producing orders of magnitude lower RNA loading into EVs compared to synthetic delivery systems (de Jong et al., 2019). Therefore, efficient and reproducible methods for loading of EVs with therapeutic RNA are currently lacking, which presents a key bottleneck for unlocking the full potential of EVs as vectors of choice for RNA delivery.

The generation of hybrid particles from EVs and lipid-based nanoparticles, including LNPs and liposomes, is emerging as the method of choice to engineer EVs for RNA delivery (Rodriguez and Vader, 2022). This is based on observations that the resulting systems merge the ‘best of both worlds', allowing the marrying of the high RNA loading property of LNPs with the highly desirable properties of EVs (low immunogenicity and superior ability to permeate biological barriers). The composite particles can therefore address the major issues associated with LNPs (Ducrot et al., 2023; Sheridan, 2023).

In an effort harness the unique properties of mEVs, namely their ability to survive the harsh gut conditions and cross the intestinal barrier (Zhang et al., 2023), our group previously generated mEV-liposome hybrid particles for oral drug delivery applications. These systems demonstrated efficient RNA loading, lower intestinal epithelial cell toxicity and superior stability compared to cationic liposomes in a simulated intestinal fluids and achieved successful siRNA transfection in J774A.1 macrophages (Zhang et al., 2024). However, to fabricate such particles we employed conditions (freeze-thawing cycles) that may be damaging to EVs or RNA. Encouraged by this work, here we report the novel engineering of mEVs into mEV-LNPs hybrids for oral delivery of siRNA. The hybrid particles were generated by microfluidics, under gentle conditions. The resulting particles were extensively characterized in terms of size, fusion efficiency and cargo loading. The systems show lower cytotoxicity than LNPs and some differences compared to mEVs and LNPs regarding cell uptake mechanisms. Importantly, hybrid particles demonstrated higher siRNA delivery efficiency (transfection) compared to LNPs, including when applied following digestion (i.e. conditions simulating oral administration). The in vivo biodistribution of RNA-loaded hybrid particles following oral administration shows some similarities to native mEVs and LNPs, as well as important differences, particularly in terms of accumulation in the colon, highlighting that, similarly to cell uptake, the biodistribution is affected by particle engineering. mEVs engineered in the manner reported here could therefore unlock the oral administration route for siRNA therapies (and possibly other types of RNA) for local delivery (to the gastrointestinal tract) or for a systemic effect.

## 2 Materials and methods

### 2.1 Materials

Bovine pasteurized skimmed milk was purchased from a local supermarket. QuantiPro™ BCA Assay Kit, Triton X-100, *N,N*-Dimethylformamide (DMF), Sodium Dodecyl Sulfate (SDS), Dulbecco's Modified Eagle's Medium (DMEM), Hank's Balanced Salt Solution (HBSS), fetal bovine serum (FBS, non-USA origin), non-essential amino acids, antibiotic/antimycotic solution, SignalSilence® Control siRNA (Cy5® Conjugate). Fasted- and Fed-State Simulated Intestinal Fluids (FaSSIF and FeSSIF, respectively) were purchased from Biorelevant (London, UK). 1,6-Diphenyl-1,3,5-hexatriene (DPH) was purchased from Cayman Chemical (Ann Arbor, MI, USA). TrypLE™ Express Enzyme, Ambion™ Nuclease-Free Water, TRIzol® reagent, Penicillin-Streptomycin, Opti-MEM™ I Reduced Serum Medium, GAPDH Activity Assay Kit (Sigma-Aldrich, Dorset, UK), Silencer™ Select GAPDH Positive Control siRNA and Silencer™ Select Negative Control siRNA were supplied by Thermo Fisher Scientific (Waltham, MA, USA). Caco-2 cells (RRID: CVCL_0025), J774A.1 murine macrophages (J774A1; RRID: CVCL_0358) and Human Embryonic Kidney 293 GFP-LC3 cells engineered to express Green Fluorescent Protein (referred to as ‘HEK293GFP’; RRID: CVCL_W347) were purchased from European Collection of Cell Cultures (ECACC, UK Health Security Agency, Salisbury, UK). Cell lines were contamination free. ExoGlow™-Protein EV Labeling Kit (Green), ExoQuick™ reagent and Exo-Check™ Exosome Antibody Array kit were purchased from System Biosciences (Palo Alto, CA, USA). 12 mm Transwell® with polycarbonate membrane inserts were purchased from Corning (Glendale, AZ, USA). Fasted- and Fed-State Simulated Intestinal Fluids (FaSSIF and FeSSIF, respectively) were purchased from Biorelevant (London, UK).

### 2.2 Cell culture

All cell lines were maintained (up to 20 passages after thawing following purchase) at 37 °C and 5 % CO_2_ atmosphere and a relative humidity of 95 %. Caco-2 and HEK293GFP cells were cultured in DMEM supplemented with 10 % *v*/v FBS, 1 % Non-Essential Amino Acids (NEAA) and 1 % v/v penicillin-streptomycin (P/S). Sub-confluent Caco-2 cultures (70–80 %) were passaged once a week using TrypLE Express (1×). J774A.1 cells were maintained in DMEM supplemented with 10 % FBS and 1 % P/S (no NEAA).

### 2.3 Isolation and characterization of EVs

Skimmed cow milk was obtained from a local supermarket. Milk EVs (mEVs) were isolated using an established three-step differential ultracentrifugation process as previously reported. Initially, 70 mL of milk was centrifuged at 13,000 ×*g* for 30 min at 4 °C using an Optima XPN-80 Ultracentrifuge equipped with a Type 45 Ti fixed-angle rotor (Beckman Coulter) to remove fats, casein and residual chymosin. The supernatant was collected and centrifuged again at 100,000 ×*g* for 50 min at 4 °C to pellet larger particles. This supernatant was then filtered through 0.2 μm filters to remove any remaining large particles. Next, the filtrate was centrifuged at 135,000 ×*g* for 90 min, yielding the mEV pellets. These pellets were washed once with sterile phosphate-buffered saline (PBS) at 135,000 ×*g* for 90 min and then resuspended in 1 mL of sterile PBS. The resuspended mEVs were stored at −80 °C for later use.

### 2.4 Preparation of siRNA-loaded Lipid Nanoparticles (LNP)

LNPs were formulated from 1,2-distearoyl-sn-glycero-3-phosphocholine (DSPC), cholesterol, 1,2-Dimyristoyl-rac-glycero-3-methoxypolyethylene glycol (DMG-PEG-2000) and the ionizable lipids heptadecan-9-yl 8-[2-hydroxyethyl-(6-oxo-6-undecoxyhexyl) amino]octanoate (SM-102) or 6-((2-hexyldecanoyl)oxy)-N-(6-((2-hexyldecanoyl)oxy)hexyl)-N-(4-hydroxybutyl)hexan-1-aminium (ALC-0315). Lipids were dissolved and kept in 100 % ethanol at a total concentration of 8 mg/mL with a molar ratio of 50 % ionizable lipids, 10 % DSPC, 38.5 % cholesterol and 1.5 % PEG-2000. This lipid ratio matches that of the Pfizer-BioNTech (ALC-0315) and the Moderna (SM-102) COVID-19 mRNA vaccine (Hald Albertsen et al., 2022). To prepare siRNA-loaded LNPs, the lipid mixture in ethanol was mixed with siRNA in 20 mM sodium acetate buffer (pH 4.0) in a microfluidic micromixer chip (Dolomite Center Ltd., Royston, UK) at a combined flow rate of 800 μL/min. The lipid-to-siRNA solution was prepared at a volume ratio of 1:3, corresponding to a flow rate ratio of 3:1 in the microfluidic chip, which was operated using a NE-300 Just Infusion Syringe Pump. Furthermore, the lipid-to-siRNA weight ratio was controlled at 30:1 to ensure optimal formulation characteristics.

### 2.5 Preparation of siRNA-loaded EV-LNP hybrids

Microfluidic micromixer chips (Dolomite Center Ltd., Royston, UK) were used to generate the hybrid particles. The isolated mEVs and siRNA-loaded LNPs were introduced into the microfluidic chips (Supporting information, Fig. S1) via infusion syringe pumps (NE-300) at a total rate of 100 μL/min (the rate ratio of the two syringe pumps was 1:1). This was followed by incubation at 37 °C for 15 min. The hydrodynamic diameter and zeta potential of the prepared siRNA-loaded hybrid particles were determined by a Zetasizer NanoZS90 instrument (Malvern Instruments, UK). For comparison, we also utilized freeze-thawing as a more established method of generating EV hybrids, which we employed recently for mEVs (Zhang et al., 2024). For this, mEVs were mixed with LNPs at different ratios and the mixtures were frozen in LN2 and thawed by sonication at 37 °C for 5 min. The cycle was repeated at least five times.

The unencapsulated siRNA and residual ethanol solution were removed by centrifugal ultrafiltration using a 100 kDa Amicon Ultra-0.5 Centrifugal Filter Unit (Merck, Dorset, UK) for 10 min at 10,000 ×g. To determine the loading efficiency, we used Alexa Fluor 647 fluorescent siRNA, which was quantified with a Tecan Infinite® 200 Pro plate reader (Tecan, Switzerland) with excitation and emission of 650 nm and 665 nm, respectively. The encapsulation efficiency of AF 647 fluorescent siRNA into hybrid particles was calculated using Eq. (1):

(1) $$\%\mathrm{encapsulation efficiency}=100\times \frac{\mathrm{amount of encapsulatedsiRNA after ultrafiltration}}{\mathrm{amount of siRNA before ultrafiltration}}\;$$

After optimization of loading fluorescently-labeled siRNA into the particles, the same approach and conditions were applied for loading different types of biologically active siRNA into EV-LNP hybrids.

### 2.6 EV-LNP fusion efficiency

To confirm the successful formation of mEV-LNP hybrids, we used Förster Resonance Energy Transfer (FRET) analysis as described previously^[18] [19]^. For this, LNPs were fluorescently labeled by incorporating a 1 % molar ratio of Nitro-2,1,3-benzoxadiazole-4-yl (NBD)-DSPE (Ex/Em: 460 nm/530 nm) and Rhodamine (Rho) Rho-PE (Ex/Em: 530 nm/588 nm) into the lipid mixture phase. Upon excitation of NBD, energy is transferred to Rhodamine via FRET, a process highly dependent on the proximity of these fluorophores. Fusion with mEVs is expected to increase the distance between NBD-DSPE and Rho-PE, leading to an increase in florescence intensity at 530 nm (NBD-PE) and a decrease at 588 nm (Rho-PE). The fluorescence emission spectra were recorded from 500 to 700 nm following excitation at 470 nm using a plate reader. The change in fluorescence would indicate fusion between LNP and mEV particles. The fusion efficiency was therefore quantified by measuring the increase in NBD fluorescence. The Max (NBD) was determined by the fluorescence intensity of NBD after lysing the vesicles with 10 μL of 2.5 % sodium dodecyl sulfate (SDS), following a two-hour incubation at 4 °C. The increase in NBD fluorescence percentage was calculated using following eq. (2):

(2) $$\mathrm{NBD}\;\mathrm{fluorescence increase}\;\left(\%\right)=\left[\mathrm{NBD}\right]/\left[\mathrm{Max}\left(\mathrm{NBD}\right)\right]\;$$

### 2.7 Characterization of mEVs and LNPs

mEVs and LNPs were characterized for particle hydrodynamic diameter (dynamic light scattering), polydispersity and surface charge (zeta-potential) using a Zetasizer Nano S (Malvern Panalytical, Malvern, UK). Samples were suspended and diluted in a suitable biological buffer to an appropriate concentration and measured in triplicates. LNP and mEV concentration were measured using nanoparticle tracking analysis (NTA) on a NanoSight NS500 instrument (Malvern, Panalytical, Malvern, UK). For NTA, samples were diluted in PBS to an appropriate particle concentration and loaded in the sample chamber. The sample was measured three times for 60 s and subsequently analyzed using the Nanosight NTA 3.4 software.

The total protein content in mEVs after isolation was evaluated using a QuantiPro™ BCA Assay Kit (Sigma-Aldrich, Dorset, UK). For characterization of mEVs, we used a commercially available Exo-Check Array kit (System Biosciences/SBI, CA, USA) to determine the expression of eight exosomal proteins (CD63, CD81, ALIX, FLOT1, ICAM1, EpCam, ANXA5 and TSG101) and GM130 cis-Golgi marker to monitor any cellular contamination.

For cryogenic transmission electron microscopy (cryo-TEM) imaging, mEVs were diluted in DI water, applied to glow-discharged Quantifoil grids, vitrified using a standard plunge-freezing procedure, and imaged on a 200 kV transmission electron microscope under cryogenic conditions. The samples were imaged at the Eindhoven University of Technology for imaging.

### 2.8 Characterization of EV-LNP hybrids

Bulk particle hydrodynamic diameter, polydispersity and surface charge of mEV-LNP hybrids were characterized as above. More detailed characterization in terms of siRNA loading and analysis of particle subpopulations following the fusion between mEVs and LNPs was undertaken using two complementary techniques, as described below.

#### 2.8.1 Nano-flow cytometry

mEVs were fluorescently labeled with ExoGlow™ Green Protein Labeling Kit (System Biosciences, USA) following the manufacturer's instructions. Cy5-conjugated siRNA (SignalSilence® Control siRNA (Cy5® Conjugate), CST #86921) was loaded into LNPs as described above, and the resulting ExoGlow-labeled EVs and Cy5-siRNA–loaded LNPs were fused into hybrid particles using the microfluidic-based method. Free unincorporated dye and unbound siRNA were removed by dialysis using a 20 kDa MWCO Slide-A-Lyzer™ MINI dialysis devices (Thermo Fisher).

Characterization by nano-flow cytometry employed a NanoAnalyzer flow cytometer (NanoFCM Inc., Nottingham, UK), equipped with dual 488 nm and 640 nm lasers and single-photon counting avalanche photodiode detectors. Manufacturer recommended laser settings were implemented with bandpass filters: 488/10 (side scatter), 525/40, and 670/30. Side scatter-based triggering was applied for detection of particles >40 nm in diameter. The trigger threshold was automatically set at three standard deviations above the mean background noise.

For each sample, a minimum of 1000 events were collected over a 1-min acquisition at a sampling pressure of 1.0 kPa to maintain an optimal event rate. Particle concentration was determined by calibrating the flow rate using 250 nm silica beads (2.17 × 10^10^ particles/mL, NanoFCM Inc., Std FL SiNP). Size distribution was estimated using a polydisperse mixture of non-fluorescent silica nanoparticles (sizes: 68, 91, 113, and 155 nm) (NanoFCM Inc., S16M-Exo). A standard curve relating side scatter intensity to particle size was generated using nonlinear regression within NanoFCM Professional V2.0 software.

#### 2.8.2 Super resolution microscopy

SM-102 lipid nanoparticles (LNPs) encapsulating negative control siRNA and hybrid nanoparticles were prepared as mentioned previously. For super resolution imaging, LNP (lipid backbone) was stained using PanLNP detection from the ONI LNP Profiler kit. The manufacturer has not disclosed the exact chemical composition of the dye, but it is optimized for LNP lipid backbone visualization and imaged in the far-red channel (647 nm). siRNA was labeled with 1c, detected in the green channel, while the mEV membrane was stained using Aco-600™ membrane dye (Acoerela), a water-soluble EV membrane-binding dye (561 nm).’ Particles were immobilized onto ONI functionalized imaging chips according to the manufacturer's instructions. The experimental workflow included chip surface preparation, LNP capture, antibody staining of surface markers, and staining of the siRNA cargo. Super-resolution imaging was performed using the NanoImager system with the Auto LNP program. Image data were analyzed via ONI's online platform CODI (https://alto.codi.bio), which enabled particle identification, quantification, and multiplexed visualization.

#### 2.8.3 Fourier Transform Infrared (FTIR) spectroscopy spectral acquisition

mEVs, LNPs, and hybrid nanoparticles were prepared as described above and subsequently rinsed in deionized (DI) water to eliminate interference from buffer components. For spectral analysis, 10 μL of each sample was applied directly onto the CaF_2_ window and air-dried to form a thin film where mEVs, LNPs or the hybrid nanoparticles are concentrated at the edge of the dried spot. A PerkinElmer Spectrum 400 FTIR microscope was used with an aperture size of 40 μm × 40 μm to obtain spectra from where the nanoparticles were concentrated. Spectral acquisition was performed at room temperature immediately after film formation. All spectra were recorded in the range of 4000–750 cm^−1^ with 64 scans. A background measurement was obtained from a clear region of the window with 128 scans. Data processing, including baseline correction, averaging, and visualization, was performed using Spectragraph software and Python-based scripts for quantitative analysis and figure generation. Protein-to-lipid (P/L) ratios were calculated by integrating the Amide *I* band (1600–1700 cm^−1^) for protein content and the CH₂/CH₃ stretching bands (2700–3040 cm^−1^) for lipid content, following the method described by Mihály et al. (Mihaly et al., 2017).

### 2.9 Cytotoxicity assay

The MTS assay [3-(4,5-dimethylthiazol-2-yl)-5-(3-carboxymethoxyphenyl)-2-(4-sulfophenyl)-2H-tetrazolium; commercially known as CellTiter 96® AQueous One Solution Assay, Promega, Madison, WI, USA] was utilized to evaluate the cytotoxicity of mEVs, LNPs, and hybrid particles in undifferentiated Caco-2 and J774A.1 cells. For this, cells were seeded into 96-well plates at a density of 10^5^ cells/mL (100 μL/well) and incubated at 37 °C in a 5 % CO_2_ atmosphere for 24 h. Following this incubation period, mEVs, LNPs, and hybrid formulations were prepared at various concentrations in Dulbecco's Modified Eagle Medium (DMEM), and the cell culture medium was replaced with these test samples. DMEM served as the negative control, while 1 % *v*/v Triton X-100 (*v*/v in Opti-MEM medium) was used as the positive control. After 48-h incubation with the test samples and controls, the cells were washed with PBS. Subsequently, 20 μL of MTS reagent was added to each well and incubated for an additional 4 h at 37 °C in a 5 % CO_2_ atmosphere. The relative metabolic activity (%) was calculated using the following Eq. (3).

(3) $$\%\mathrm{Relative metabolic activity}=100\times \frac{S-T}{\mathrm{Negative}-T}\;$$

where ‘S' represents the absorbance of the sample, ‘T' represents the absorbance of the Triton X-100 treated group, and ‘Negative’ represents the absorbance of the non-treatment (culture medium) group.

### 2.10 Cell uptake of siRNA-loaded mEVs and EV-LNP Hybrids

*Aco-600™* labeled hybrids were diluted in HEPES buffer (to 0.1 mg/mL) and applied to the apical chamber of differentiated Caco-2 cell monolayers for 3 h at 37 °C. Cells were then washed three times with PBS to remove unbound particles and fixed with 4 % paraformaldehyde for 15 min. Uptake was visualised by confocal microscopy (Leica TCS SP8).

### 2.11 Epithelial transport of siRNA-loaded mEVs and EV-LNP hybrids

Caco-2 cells were cultured on 12-well polycarbonate Transwell inserts in DMEM for 21 days to produce differentiated intestinal epithelial monolayers. Transepithelial electrical resistance (TEER) was measured using an EVOM voltohmmeter (World Precision Instruments, Sarasota, FL, USA) to ensure monolayer barrier integrity. Prior to the transport study, the culture medium was replaced with Hanks' Balanced Salt Solution (HBSS) and incubated for 45 min for equilibration. We then applied to the apical side one of the following samples: 500 μL of mEVs loaded with fluorescent siRNA (Cy-5 conjugate, Cell Signaling Technology, MA, USA) by electroporation, EV-LNP hybrids prepared via the freeze-thaw method, EV-LNP hybrids generated by microfluidics, or control fluorescent siRNA. Samples were applied at 0.05 mg/mL (protein concentration of the mEVs based on the BCA kit assay) in HBSS. Cell monolayers were incubated with the samples for 3 h. During the incubation, 100 μL of the basolateral solution was collected regularly (at 30 min intervals), with the sampled solution replaced with HBSS to maintain sink conditions and a constant basolateral volume. The fluorescent siRNA loaded samples were quantified using a Tecan Infinite® 200 Pro fluorescence plate reader at excitation and emission of 640 nm and 680 nm, respectively.

### 2.12 Determination of nanoparticle cell uptake pathways

Caco-2 cells were seeded onto 0.4 μm pore-size Transwell inserts (polycarbonate membrane) and cultured for 21 days to allow formation of a confluent, polarized monolayer that mimics the intestinal epithelial barrier. Medium was changed every other day, and monolayer integrity was confirmed by TEER measurements prior to the experiment. To investigate nanoparticle uptake pathways, pharmacological inhibitors targeting specific endocytic mechanisms were employed. Cells were pre-treated for 30 min at 37 °C in Hank's Balanced Salt Solution (HBSS) containing one of the following inhibitors: dynasore (20 μg/mL), genistein (50 μg/mL), 5-(N-ethyl-N-isopropyl)-amiloride (EIPA, 10 μg/mL), nocodazole (12.5 μg/mL), chlorpromazine (20 μg/mL), or methyl-β-cyclodextrin (MβCD, 6650 μg/mL). After incubation, inhibitors were removed, cells were washed with PBS, and fluorescently labeled samples were added to the apical chamber in fresh HBSS containing the same inhibitor concentrations. Control samples were added without inhibitors. After 4 h, cells were washed, lysed, and fluorescence was quantified using a microplate reader to assess internalization efficiency. Data were expressed as percentage uptake relative to control (no inhibitors) and analyzed statistically.

### 2.13 Transfection experiments

#### 2.13.1 GAPDH siRNA-loaded LNPs and EV-LNP hybrids

Caco-2 cells were seeded in 96-well plates at 5000 cells per well and cultured for 24 h. GAPDH siRNA-loaded EV-LNP hybrids or siRNA-loaded LNPs were diluted in Opti-MEM Medium to 0.1 nmol/mL and incubated with cells for 5 h at 37 °C in a 5 % CO_2_ atmosphere. Subsequently, the medium was replaced with complete growth medium and the cells were incubated for an additional 43 h. Following incubation, GAPDH activity was measured using a GAPDH Activity Assay kit (Sigma-Aldrich, Dorset, UK) according to the manufacturer's instructions. siRNA alone served as negative control, while GAPDH siRNA transfected with a commercial transfection reagent (Lipofectamine™ CRISPRMAX™) was used as the positive control. The percentage of remaining GAPDH gene expression was calculated with Eq. (4):

(4) $$\%\mathrm{remaining expression}=100\times \frac{∆450-\mathrm{GAPDH}}{∆450-\mathrm{Negative}}\;$$

#### 2.13.2 Anti-GFP siRNA-loaded hybrids

In addition to Caco-2 cells, HEK293GFP cells were also used to evaluate the siRNA gene silencing efficiency of the EV-LNP hybrids. These were seeded in 96-well plates at 5000 cells per well. After 24 h, cells were transfected with various concentration of hybrid particles loaded with anti eGFP siRNA diluted in DMEM medium. Cells were continuously monitored for 48 h using an AutoLCI automatic live-cell imaging system (World Precision Instruments, Sarasota, FL, USA). Images were captured every 8 h using a 4× objective lens under both bright field and green fluorescence conditions. This approach allowed for real-time observation of cell growth and changes in the expression of GFP. ImageJ software was used to semi-quantify the generated images. Lipofectamine CRISPRMAX™ Transfection Reagent was used as a positive control with siRNA concentration of 50 nM, according to the manufacturer's protocol. Cells incubated with 30 nM eGFP siRNA were used as the negative control.

#### 2.13.3 Effect of simulated intestinal fluids on transfection efficiency of LNPs and hybrids

Fasted State Simulated Intestinal Fluid (FaSSIF, pH 6.5) and Fed State Simulated Intestinal Fluid (FeSSIF, pH 5.0) were employed as commercially available simulated intestinal digestive fluids which an oral delivery system is expected to encounter in the intestine before interacting with epithelial cells. Therefore, here we determined whether EV-LNP hybrids can protect the siRNA therapeutic payload in these environments. For these experiments, 100 μL aliquots of 0.05 mg/mL EV-LNP hybrids were mixed with 400 μL of FaSSIF or FeSSIF and incubated at 37 °C for 1.5 h under continuous stirring at 40 rpm. Following the digestion process, nanoparticles were isolated via centrifugal ultrafiltration using a 100 kDa Amicon Filter Unit through four sequential centrifugation steps, each conducted at 10,000 *g* for 10 min. To ensure the removal of residual debris from the digestion solutions, 500 μL of HEPES buffer was introduced between each centrifugation step. The resulting digested nanoparticles were then resuspended in 60 μL PBS and incubated with Caco-2 cells for 48 h. Thereafter, the expression level of the GAPDH protein in the cells was measured by a commercial kit as previously described.

### 2.14 In vivo biodistribution study

Animal studies were carried out under the University of Prishtina (Kosovo) guidelines for the care of experimental animals and were approved by Research and Ethics Committee of the Faculty of Medicine (reference number 2692). Male Balb/c white mice weighing 25-30 g were housed under controlled conditions (12-h light/dark cycle, 20–24 °C, 40–60 % humidity) in Digitally Ventilated Cages (DVC, Tecniplast, Italy), with ad libitum access to food and water. Mice were individually weighed prior to study protocol to ensure accurate dosing. EV-LNP hybrids were labeled fluorescently using one of two different fluorophores, which enabled us to track the carriers, as well as the loaded siRNA. To achieve this, LNPs and EV-LNP hybrids loaded with GAPDH siRNA were labeled with DiR or near-infrared IRDye800CW siRNA, respectively. OptiPrep density gradient centrifugation was performed on the labeled formulations to confirm that the dye was detectable only when bound to the nanoparticle membrane, with a validated density range of 1.08–1.22 g/mL. EZ-Anesthesia device was used to administer 3 L/min of isoflurane for induction of anesthesia and 1.5 L/min for maintenance. A total of 700 μL of the designated formulation (concentration of 4.15 × 10^7 particles/mL) was administered through oral gavage (divided in two intervals,10 min apart) to each mouse and the animals were observed for signs of distress or discomfort in the post administration phases. Afterwards, imaging was performed with Pearl Trilogy imaging equipment (LI-COR, USA) in different time set points (2, 4, 8, and 24 h) post administration to determine the biodistribution of each nanoformulation. Imaging was captured with Image Studio Software (version 5.5) using scan resolution of 170 μm, focus position 0. The detection of fluorescently labeled formulations was done using an 800 nm Channel Laser Source (Excitation 785 nm; Emission 820 nm). The organs of interest consisted of the gastrointestinal tract, liver, spleen, kidneys, heart and lungs. After 24 h, mice were euthanized using a Ket-A-Xyl cocktail, and major organs were harvested for further imaging and analysis.

In a separate set of experiments, for the detection of the RNA payload incorporated into the hybrid nanoparticles, near-red IRDye® 800 CW siRNA was employed and we carried out 8 h post administration sacrifice due to the stronger fluorescence signal.

Data was analyzed by measuring the fluorescence signal (intensity) by drawing the region of interest. Biodistribution data from several time points were compared using statistical analysis, using GraphPad Prism. Data are presented using the mean ± standard deviation (SD) using at least three technical replicates and repeated experiments. Statistical analysis was done by unpaired Student's *t*-test or ANOVA.

## 3 Results and discussion

### 3.1 Characterization of EVs and siRNA-loaded LNPs

Table S1 and Fig. S2 (Supporting Information) show the hydrodynamic diameter (determined by NTA), yield, protein concentration and surface charge of the isolated mEVs. The hydrodynamic diameter (∼150 nm) and zeta-potential (∼10 mV) of mEVs falls within the range of those reported previously (Zhang et al., 2024). The detection of mEV protein markers, identified using Exo-Check Array (Fig. S2B), shows that mEVs expressed markers including CD63, CD81, Intercellular Adhesion Molecule-1 (ICAM-1), ALG-2- interacting protein X (ALIX; cytosolic proteins)，Flotillin 1 (FLOT1), Annexin A5 (ANXA5) and Cis-golgi matrix protein (GM130). The expression of epithelial cell adhesion molecule (EpCAM) and Tumor Susceptibility Gene 101 (TSG101) was not apparent. The detection of a very faint positive band for the Golgi protein GM130 (Cis-golgi matrix protein) suggests the possibility of minor intracellular protein contamination and similar observations have been reported previously (Kim et al., 2022; Kupsco et al., 2021; van Herwijnen et al., 2016). The spherical morphology and bilayer membrane structure of mEV was further confirmed by cryo-TEM) (Supporting Information, Fig. S2C).

The characterization of siRNA-loaded LNPs based on two different ionizable lipids, SM-102 and ALC-0315, is shown on Table S2 (Supporting Information). Particles had hydrodynamic diameters of around 160 nm, with a PdI < 0.2 and zeta potential in the range of 33.5–46.5 in sodium acetate buffer (pH 4.0). The siRNA entrapment efficiency was 90 % and 85 % for LNPs containing SM-102 and ALC-0315 lipids, respectively.

### 3.2 Confirmation of fusion between LNPs and mEVs

**Fig. 1:**
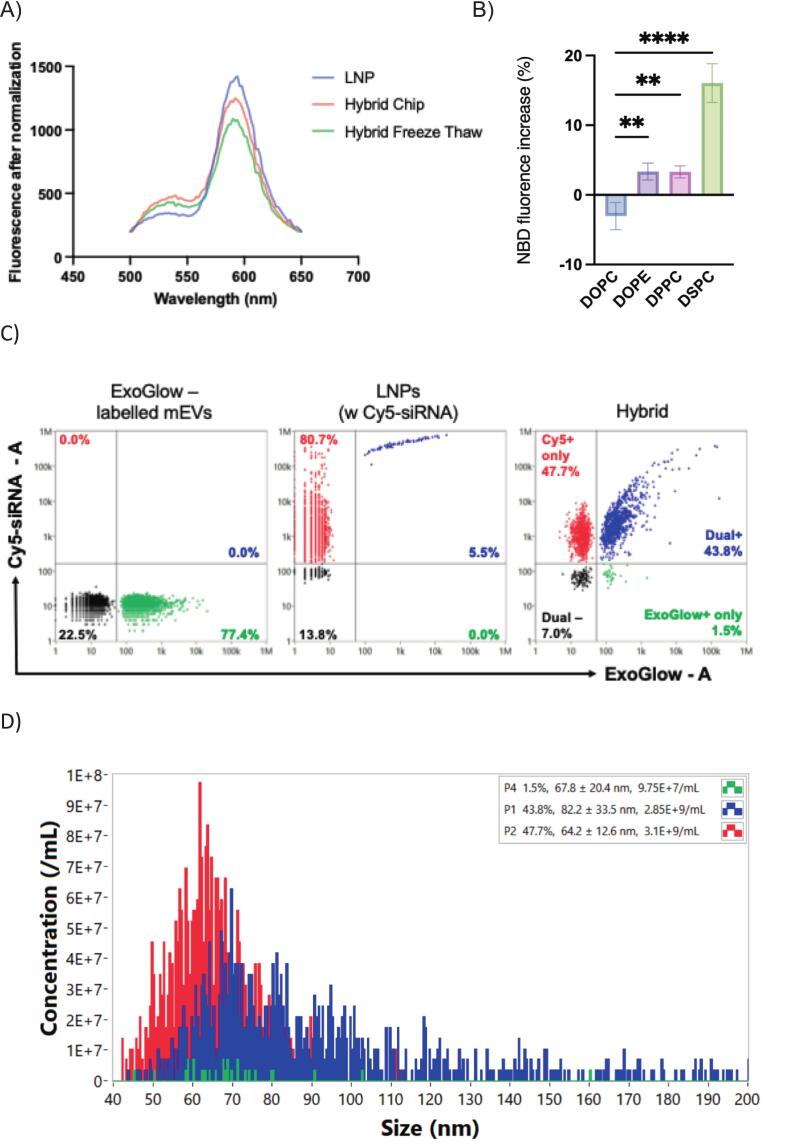
Fusion and characterization of mEV–LNP hybrid nanoparticles. A) Fluorescence resonance energy transfer (FRET)-based determination of membrane fusion of mEVs and LNPs. Fluorescence spectra of LNPs and hybrid particles prepared by two separate methods—five cycles of freeze–thawing or microfluidic mixing (‘chip’)—are shown. Recovery of fluorescence at 534 nm, accompanied by a reduction at 590 nm, indicates successful fusion of LNPs with mEVs. Fluorescence intensity values were normalized using Python. B) Fusion efficiency of hybrid particles expressed as the percentage increase in NBD fluorescence (y-axis). Data represent mean ± SD (n = 3). Statistical analysis conducted using one-way ANOVA, *p < 0.05, **p < 0.01, ***p < 0.001. C) Representative nano-flow cytometry (nFCM) plots showing Cy5-siRNA–loaded LNPs (red), ExoGlow-labeled mEVs (green), and EV–LNP hybrids. Dual-positive particles (ExoGlow^+^/Cy5^+^) correspond to successfully fused hybrids. Data are presented as mean ± SD (n = 3 independent experiments). D) Particle size distribution profiles of EV–LNP hybrids (blue), parental LNPs (red), and mEVs (green) obtained by nFCM.

The spectral change, shown in Fig. 1, confirmed the successful membrane fusion between LNPs and mEVs. To enable this experiment, LNPs were doped with a FRET pair of dyes, namely Rho and NBD. Fig. 1A shows that fluorescence intensity at around 534 nm increased and fluorescence intensity at 583 nm decreased after the fusion of mEVs and LNPs via microfluidic micromixing and freeze-thawing. To screen the effects of different helper lipids on the fusion efficiency between mEVs and LNPs, FRET was used to indicate particle fusion, as shown in Fig. 1B. Fusion of the labeled LNPs with unlabeled mEV was detected as an increase in NBD fluorescence (Parlati et al., 1999). The neutral lipid, DSPC, was selected as the base component of the screened LNP formulations as it showed the highest fusion efficiency among the four commonly used helper lipids (Sato et al., 2016; Zhang et al., 2022).

### 3.3 Characterization of EV-LNP hybrids

Table 1 shows the physicochemical characteristics of siRNA-loaded EV-LNP hybrids generated by microfluidics, providing a comparison with counterparts fabricated using the freeze-thawing method. The data shows that the hydrodynamic diameter, PdI and surface charge of the hybrids dependent upon the LNP:EV ratio. The freeze-thaw method produced particles with the smallest diameter and lowest PdI at LNP:EV ratios of 5:1 and 1:1, respectively. On the other hand, the microfluidic method generated hybrid particles with the smallest diameter and PdI at 1:1 LNP:EV ratio. Importantly, out of the two methods used to prepare hybrid EV-LNP particles, the microfluidic preparation method produced hybrid particles with a combination of acceptable PdI and size closest to native EVs at 1:1 LNP:EV ratio (133 nm; PdI 0.19). To further assess the effect of fabrication methods on particle size, we compared the median diameters of SM-102 LNPs, hybrid nanoparticles, and milk-derived EVs prepared via microfluidic mixing ('hybrid chip') or freeze–thawing ('hybrid and F-T') (Fig. S3, Supporting Information). The data show that microfluidic-generated hybrids displayed the smallest and most uniform size distribution, supporting its suitability for EV-LNP hybrid fabrication.

**Table 1:** Characterization of siRNA-loaded hybrid mEV-LNP particles.

Ratio		5:1	1:1	1:5
Freeze-thaw method	Hydrodynamic diameter (nm)	166.4 ± 3.47	193.6 ± 2.95	285.0 ± 3.32
	PDI^a)^	0.257 ± 0.015	0.194 ± 0.016	0.359 ± 0.044
	ζ-potential (mV)	7.944 ± 0.28	−11.58 ± 0.99	−10.92 ± 0.64
	Hydrodynamic diameter (nm)	158.4 ± 2.37	133.1 ± 1.84	168.1 ± 3.11
Microfluidics	PDI^a)^	0.419 ± 0.05	0.19 ± 0.016	0.276 ± 0.019
	ζ-potential (mV)	8.765 ± 0.16	−10.6 ± 0.98	−12.25 ± 0.96

Formulations were prepared by liquid nitrogen (LN2) freeze-thaw cycles and microfluidic mixing with different LNP to milk extracellular vesicles (mEVs) ratio (based on the particle concentration determined by nanoparticle tracking analysis). ^a^PdI: polydispersity index. Data shown as the mean ± SD (n = 3).

To further resolve particle heterogeneity and confirm structural integration of mEV and LNP components, we employed dual-fluorescent nano-flow cytometry (nFCM) (Fig. 1C). mEVs were labeled with ExoGlow™ Green, and Cy5-conjugated siRNA was pre-loaded into LNPs prior to hybridization. After dialysis to remove free dye, samples were analyzed on a NanoAnalyzer equipped with 488 nm and 640 nm lasers. Only 1.5 % of particles were ExoGlow^+^ alone, whereas 45–50 % were dual-positive for ExoGlow and Cy5, indicative of efficient hybrid formation. Moreover, the presence of dual-positive particles reflects not only membrane-level interactions but also true structural integration with co-loading of both membrane components and cargo, thereby achieving cargo co-delivery. Representative nFCM dot plots of each sample are shown in Supporting Information, Fig. S5.

Particle size distribution obtained by nFCM demonstrated that hybrid nanoparticles (median size 66–71 nm) were intermediate between parental mEVs (63–66 nm) and LNPs (78–82 nm), with no evidence of large aggregates (Fig. 1D). Specifically, statistical analysis (Kruskal-Wallis test with Dunn's post-hoc) revealed that mEV and LNPs are significantly different in size, while EV–LNP hybrids are not significantly different to either. These findings were corroborated by super-resolution imaging, which confirmed colocalization of mEV and LNP markers at the single-particle level (Fig. S4, Supporting Information).

Together, these results demonstrate that microfluidic fusion produces EV–LNP hybrids that retain mEV-like dimensions while enabling efficient co-loading of membrane components and siRNA cargo, with improved reproducibility over conventional freeze–thawing.

#### 3.3.1 FTIR spectroscopic analysis of hybrids

**Fig. 2:**
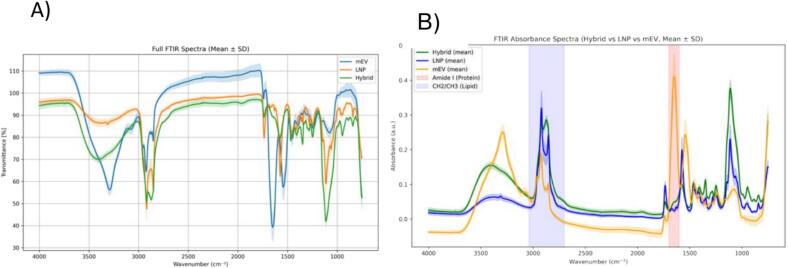
Representative FTIR spectra and absorbance spectra for milk-derived extracellular vesicles (mEVs), lipid nanoparticles (LNPs), and their hybrid constructs. A) Overlay of the full FTIR spectra revealed distinct yet partially overlapping profiles among mEV mEV (blue), LNP (orange), and Hybrid samples (green). B) Corresponding spectra displayed in absorbance units, shown as mean (solid lines) ± SD (shaded regions). The Amide *I* region (1600–1700 cm^−1^, protein, highlighted in red) and the CH₂/CH₃ stretching region (2700–3040 cm^−1^, lipid, highlighted in blue) were used for numerical integration to calculate the spectroscopic protein-to-lipid (P/L) ratio. (For interpretation of the references to colour in this figure legend, the reader is referred to the web version of this article.)

FTIR absorbance spectra distinguished mEVs, LNPs, and hybrid nanoparticles by their unique molecular signatures (Fig. 2A).

The full-spectrum comparison and focused analysis on the amide I region (Fig. 2B) confirmed mEVs exhibited a prominent peak at ∼1650 cm^−1^, characteristic of protein-rich vesicles. In contrast, LNPs showed intense CH₂ symmetric and asymmetric stretches (2850 and 2920 cm^−1^) and a sharp ester carbonyl band at ∼1735 cm^−1^, highlighting their synthetic lipid nature. Further detailed comparisons revealed that the hybrid spectrum partially overlapped with that of LNPs, but displayed unique features: a shoulder at ∼2870 cm^−1^ and broadening of the symmetric PO₂^−^ stretching band at ∼1120 cm^−1^, both absent in the individual components. These changes suggest a possible reorganization of the lipid environment and support membrane fusion rather than simple mixing. FTIR spectra of DI-water background was subtracted during acquisition. Exported spectra (in transmittance, %T) were converted to absorbance using $A=-\log_{10}\left(\frac{T}{100}\right)$. Wavenumbers were sorted in ascending order to ensure consistent numerical integration. The Amide *I* region (1600–1700 cm^−1^) was integrated to obtain the protein-related area $A_{\mathrm{Amide}\;I}$; the C—H stretching region (2700–3040 cm^−1^, CH₂/CH₃, including the olefinic band) was integrated to obtain the lipid-related area $A_{CH_{2}/CH_{3}}$The spectroscopic protein-to-lipid ratio for each replicate was calculated as

$$P/L=\frac{A_{\text{Amide}\;I\;\left(1600-1700\;cm^{-1}\right)}}{A_{{\mathrm{CH}}_{2}/{\mathrm{CH}}_{3}\;\left(2700-3040\;cm^{-1}\right)}}$$

Protein-to-lipid (P/L) ratios calculated from FTIR absorbance spectra (Table 2) further supported these findings. mEVs exhibited a high P/L ratio (1.335 ± 0.26), consistent with their protein-rich profile, while LNPs had a low ratio (0.09 ± 0.009), reflecting minimal protein content. Hybrid nanoparticles yielded an intermediate P/L ratio (0.14 ± 0.003), indicating partial incorporation of mEV-associated proteins into a lipid-dominated structure. Notably, the ester carbonyl band also appeared in hybrids, confirming the integration of LNP-like lipid species. Taken together, these spectral and compositional characteristics provide strong evidence that the hybrid nanoparticles result from structural integration through lipid fusion and not passive mixing of mEVs and LNPs.

**Table 2:** Protein-to-lipid (P/L) ratios calculated from FTIR absorbance spectra.

Sample Type	Mean P/L Ratio	Standard Deviation	n
mEV	1.335	±0.151	12
LNP	0.083	±0.009	8
Hybrid	0.14	±0.003	6

The advantages of microfluidics over the freeze-thaw methods of achieving the fusion between mEVs and LNPs are clear. In non-disruptive microfluidic conditions of laminar flow, the two particle populations experience repeated, close and sustained interfacial contact that bulk incubation cannot provide, facilitating efficient fusion, as demonstrated by our detailed characterization (specifically a high fraction of dual-positive hybrids observed with nFCM). By contrast, conventional disruptive methods (e.g., freeze–thaw/sonication) generate lower fusion efficiency (and consequently lower cargo loading), while potentially compromising the delivery performance by perturbing membrane proteins and lipid organization.

### 3.4 Effect of LNPs and EV-LNP hybrids on cell viability

**Fig. 3:**
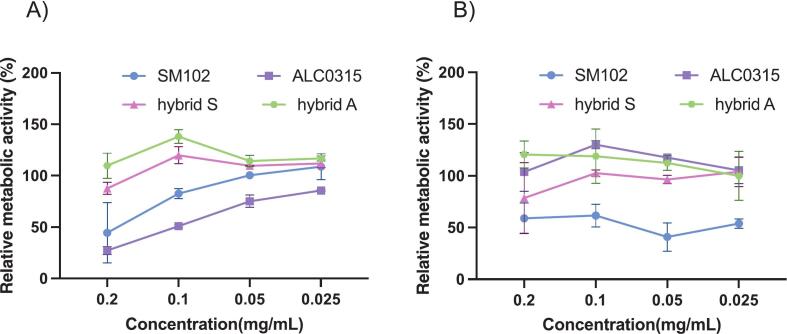
Cytotoxicity of mEV-LNP hybrids in Caco-2 (A) and J447A.1 (B) cells. Metabolic activity was evaluated by the MTS assay. Cells were treated with different concentration (0, 0.2, 0.1, 0.05, 0.025 mg/mL) of LNPs and hybrids for 48 h in complete growth medium. Data are presented as mean ± SD from 3 independent experiments (*n* = 3).

To compare the cytotoxicity profile of LNPs (based on two different ionizable lipids, SM-102 and ALC-0315) and EV-LNP counterparts, we used two different cell lines, namely intestinal epithelial Caco-2 cells and murine macrophage J447A.1 cells, and a common metabolic activity assay. The data in Fig. 3A shows that both types of LNPs showed a concentration-dependent toxicity in Caco-2 cells, with the higher concentration ranges showing a significant cytotoxicity. In contrast, except for the highest concentration of EV-LNP hybrids based on SM-102 with which there was around 20 % reduction in relative metabolic activity, both types of EV-LNPs showed a non-toxic profile in the concentration range tested. In J447A.1 cells (Fig. 3B) the picture was less clear, with SM-102 LNPs showing significant toxicity and ALC0315 LNPs not exhibiting any statistically significant toxicity at all concentrations. However, apart from the highest tested concentration of EV-LNP hybrids with SM-102 lipid, which was associated with approximately 25 % reduction in metabolic cell activity, other EV-LNPs with this lipid did not show toxicity in this cell line. Overall, cell toxicity results reveal a favourable profile of EV-LNP hybrid particles compared to LNPs with the same ionizable lipid.

### 3.5 Intestinal epithelial cell uptake and internalization pathways

**Fig. 4:**
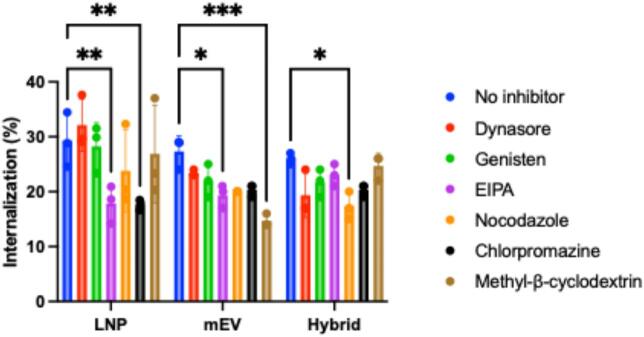
Effect of endocytosis inhibitors on the uptake of LNPs, mEVs, and hybrid nanoparticles in differentiated Caco-2 monolayers. Caco-2 monolayers were treated with fluorescently labeled LNPs, mEVs, or hybrid nanoparticles in the absence or presence of various pharmacological inhibitors of endocytosis. Uptake efficiency was quantified by fluorescence intensity, normalized to the untreated (no inhibitor) control group. Data are presented as mean ± SD (*n* = 3). Statistical significance was determined using one-way ANOVA with post hoc tests: *p < 0.05, **p < 0.01, ***p < 0.001.

Fig. 4 shows the effect of a panel of pharmacological inhibitors of endocytosis on the uptake of mEVs, LNPs and hybrid particles in differentiated (Transwell-cultured) Caco-2 cells. All systems were internalized by polarized Caco-2 cells via active endocytic pathways, as indicated by reduced uptake in the presence of inhibitors. LNP uptake was most strongly inhibited by EIPA and chlorpromazine, suggesting a primary reliance on micropinocytosis and clathrin-mediated endocytosis. mEV uptake was significantly attenuated by EIPA and methyl-β-cyclodextrin (MβCD), indicating involvement of macropinocytosis and lipid raft–mediated pathways. Finally, only nocodazole, a microtubule depolymerizing drug, influenced the cell uptake of EV-LNP hybrid particles. Fig. S6 (Supporting Information) confirms the uptake of hybrid particles in Caco-2 cells by confocal microscopy.

*Literature suggests that LNPs* can be internalized by multiple mechanisms, including micropinocytosis, clathrin-mediated and caveolae-mediated endocytosis (Hou et al., 2021). EVs in general are predominantly internalized through classical endocytosis mechanism (Gandek et al., 2023). Interestingly, comparing the effect of pharmacological inhibitors on the uptake of bovine mEVs with that on human mEVs, reported in a recent study by our group (Luo et al., 2025), the two sources of mEVs appear to display different responses to the pharmacological inhibitors since the Caco-2 uptake of human mEVs was reduced by chlorpromazine, nocodazole and dynasore. To our knowledge, a comparison of cellular uptake pathways of EVs with EV-LNP hybrids, as carried out here, has not been reported previously. Our results show that hybrid nanoparticles display a different sensitivity to pharmacological endocytosis inhibitors compared to the EVs and LNPs from which they originate, suggesting that the engineered hybrid particles utilize alternative endocytosis pathways (in Caco-2 cells). It must be noted that the experimental setup employed here to test the effect of endocytosis inhibitors, namely differentiated and polarized Caco-2 monolayers, is more physiologically relevant compared to the use of non-differentiated cells, which are typically employed in the literature. The importance of the use of differentiated epithelial cells in cell uptake studies is highlighted in a recent study, which demonstrated clear differences in endocytic processes used by intestinal epithelial Caco-2 cells, depending on their differentiation status (Bannunah et al., 2024).

### 3.6 Intestinal epithelial transport of siRNA-loaded EV-LNP hybrids

Microfluidics-fabricated siRNA-loaded EV-LNP hybrids bearing the ionizable lipid SM-102 were tested for intestinal epithelial transport in the established Caco-2 intestinal model. We compared the epithelial translocation of hybrid particles with that of mEVs loaded with siRNA by electroporation, as well as hybrid systems generated via the freeze-thaw method. Fig. S7 (Supporting Information) shows that around 2 % of the applied siRNA within microfluidics-engineered hybrids transported across the Caco-2 monolayers in three hours. This was similar to siRNA loaded into mEVs by electroporation and significantly higher than the siRNA in hybrid EV-LNP particles formulated by freeze-thawing and siRNA alone. This highlights the potential of the systems engineered in this work for intestinal delivery of therapeutic RNA. The comparison with hybrid EV-LNP particles fabricated by freeze-thawing is particularly interesting. We previously demonstrated the formulation of similar systems based on a cationic lipid (non-ionizable) and observed a similar level of transport across Caco-2 monolayers (Zhang et al., 2024). The RNA-loaded hybrid EV-LNP particles developed here demonstrate a more efficient intestinal epithelial transport, which may be explained by more desirable physicochemical characteristics (e.g. smaller size) of the hybrid particles, or the use of gentler conditions to facilitate particle fusion, potentially minimizing any structural damage to the particle components (assumed to be within EVs) that facilitate interaction with and transport across the intestinal epithelium.

While at this stage it is not possible to fully define the clinical application and specific target of the hybrid systems, the intestinal epithelial transport data preliminarily indicate that the EV-LNPs transport across the intestinal epithelium for RNA payload delivery to subepithelial blood supply and beyond. Interestingly, epithelial transport of hybrid particles was similar to mEVs loaded with siRNA by electroporation. It must be noted that a direct comparison of these two groups is challenging to undertake since the percentage of particles containing siRNA, as well as the amount of siRNA contained within each particle is expected to be different in the two groups. It is also important to note that in previous work we have reported a higher intestinal epithelial transport of mEVs (of over 10 % in 90 min) (Zhang et al., 2023). The lower value of 2 % (in three hours) with the hybrid particles observed here is likely to relate to the quantitation of siRNA rather than EVs.

### 3.7 Transfection efficiency of EV-LNP hybrids

#### 3.7.1 GAPDH siRNA transfection in Caco-2 cells

**Fig. 5:**
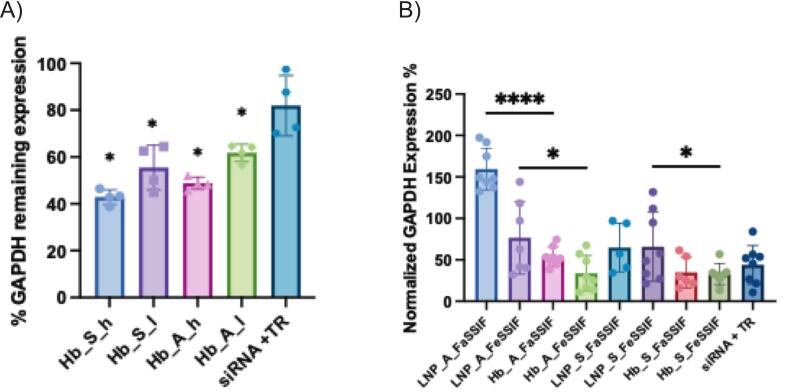
Effect of mEV-LNP hybrid nanoparticles on GAPDH transfection in Caco-2 cells. A) GAPDH protein expression levels in Caco-2 cells transfected with GAPDH siRNA-loaded hybrid nanoparticles. The hybrids were synthesized via microfluidic methods using two distinct lipid nanoparticle (LNP) formulations, SM-102 and ALC-0315. The concentration of mEVs and hybrids was 0.08 mg/mL(h) or 0.04 mg/ml(l) and corresponding to the siRNA concentration of 0.1 nmol/mL. siRNA was transfected by application of the samples diluted in OPTI-MEM medium for 5 h, followed by replacement with growth medium. B) GAPDH expression levels in Caco-2 cells after transfection with GAPDH siRNA-loaded lipid nanoparticles (LNP) and hybrid nanoparticles (Hb), formulated using ALC-0315 (A) and SM-102 (S). Nanoparticles were treated with fasted state and fed state simulated intestinal fluids (FaSSIF and FeSSIF, respectively), prior to transfection. The Lipofectamine CRISPRMAX Transfection Reagent (TR) was used as positive control**** indicates *p* < 0.0001. * indicates *p* < 0.05. Data are shown as the mean ± SD.

To probe the oral siRNA delivery potential of EV-LNP hybrids, we undertook a series of transfection experiments. We initially tested the GAPDH transfection efficiency of the hybrid formulations in intestinal epithelial Caco-2 cells. The data in Fig. 5A generally show that EV-LNP hybrids generated from both ionizable lipids (and applied at two different concentrations for five hours) were capable of reducing GAPDH expression to a significantly larger extent than a commercially available transfection reagent. The reduction of GAPDH expression amounted to approximately 40–60 %, with the effect on GAPDH expression being significantly larger for hybrid EV-LNP particles compared to the commercial transfection reagent control. It must be noted that the intestinal Caco-2 cell line is known to be resistant to transfection (Clayburgh and Turner, 2005), which explains the paucity of transfection data in this cell line and prevents a comparison of the performance of hybrid mEVs with more established siRNA delivery systems.

To achieve gene silencing following oral administration, the delivery system should be capable of surviving the hostile biochemical environment of the gastrointestinal tract. Whilst this includes the low pH of the stomach, we were not interested in determining the stability of the formulations in stomach biofluid since the reasonable assumption is that an advanced oral formulation, such as the RNA delivery systems proposed here, would be administered orally within a suitable enteric coated solid dosage form. However, we tested the stability of the EV-LNP hybrids in intestinal biofluids. This is because of the widely reported instability of lipid bilayer-based liposomes in the intestinal fluid because of their interaction with bile salts (Bohsen et al., 2023; Jash et al., 2021). We therefore utilized fasted- and fed-state simulated intestinal fluids (containing different concentrations of bile salts) and exposed the EV-LNP hybrids in these fluids prior to testing their transfection efficiency. The results in Table S3 (Supporting Information) show the effect of treatment with simulated intestinal fluids on particle hydrodynamic diameter, demonstrating a statistically significant increase for mEVs and hybrids following treatment with both types of fluids. Fig. 5B show that the hybrid formulations can preserve the biological activity of the encapsulated siRNA, since, following a 1.5-h exposure to digestion conditions, the hybrid nanoparticles were able to successfully transfect intestinal Caco-2 cells. However, additional studies are required to further assess the activity of the EV-LNP hybrids following exposure to more realistic digestive conditions, including the presence of various digestion related enzymes. When comparing with LNPs, EV-LNP hybrids demonstrated a significantly higher transfection efficiency following exposure to simulated intestinal fluids. This is the first time that such an important finding is reported. The data potentially indicates the superiority of the hybrid formulations to LNPs for oral RNA delivery and attribute the resistance of EV-LNP hybrids to digestive conditions to the properties of naïve mEVs, which are known to be stable against digestion and capable of being absorbed into the systemic circulation (Samuel et al., 2021).

The transfection efficiency was also confirmed in a different cell line, against a different siRNA target, specifically GFP-expressing HEK293. The results (Supporting Information, Fig. S8) clearly show that the fluorescence intensity of the cells is reduced following the incubation with hybrid nanoparticles, indicating that both hybrid EV-LNP systems effectively silenced GFP expression. Interestingly, in HEK293GFP cells, the gene silencing efficiency of siRNA was considerably lower than that of commercially available transfection reagent control, which is a finding that is not mirrored in Caco-2 cells (where the same transfection reagent was used). The greater transfection efficacy of the EV-LNP hybrid nanoparticles generated here in intestinal epithelial cells compared to HEK293 cells is an interesting observation and it may possibly be attributed to a cell-dependent uptake of the systems, with hybrid particles possibly being preferentially taken up by intestinal epithelial cells. This may be related to the physiological function of mEVs as potential messengers of biological signals to the gut and any associated ability to be internalized into intestinal epithelial cells (Luo et al., 2025). However, this remains to be confirmed in the future.

### 3.8 In Vivo Biodistribution

While the biodistribution of mEVs following oral administration has previously been investigated (Samuel et al., 2021), to our knowledge there are no prior studies of the biodistribution of any type of orally-administered EV-LNP hybrid nanoparticles loaded with siRNA. We therefore for the first time examined the biodistribution of EV-LNP hybrids following oral administration, providing a comparison with the biodistribution of its constituent particles (mEVs and LNPs). Given the known issues with potential experimental artefacts with lipid fluorescence dyes used to label mEVs and LNPs (and in our case, hybrids), we performed the biodistribution experiment under two separate conditions, with one condition employing particles (hybrids, mEVs and LNPs) labeled with the membrane dye DiR and another employing mEV-LNP hybrids and LNPs loaded with fluorescently-labeled siRNA (IRDye®800CW siRNA).

**Fig. 6:**
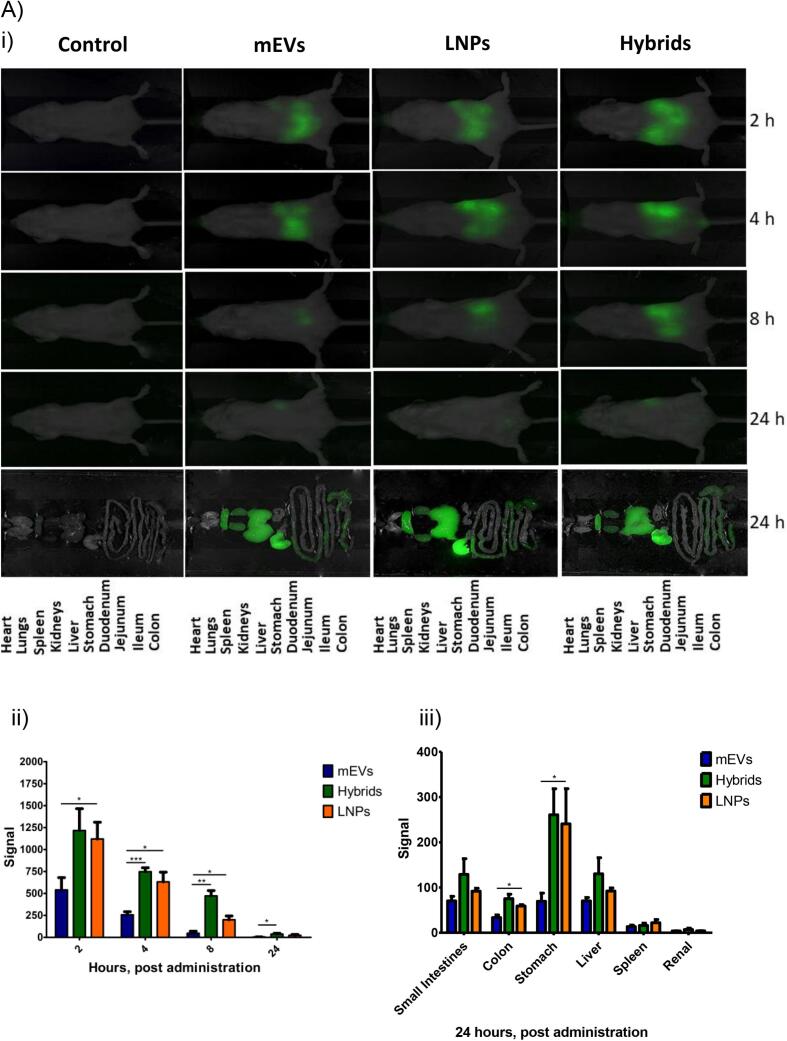

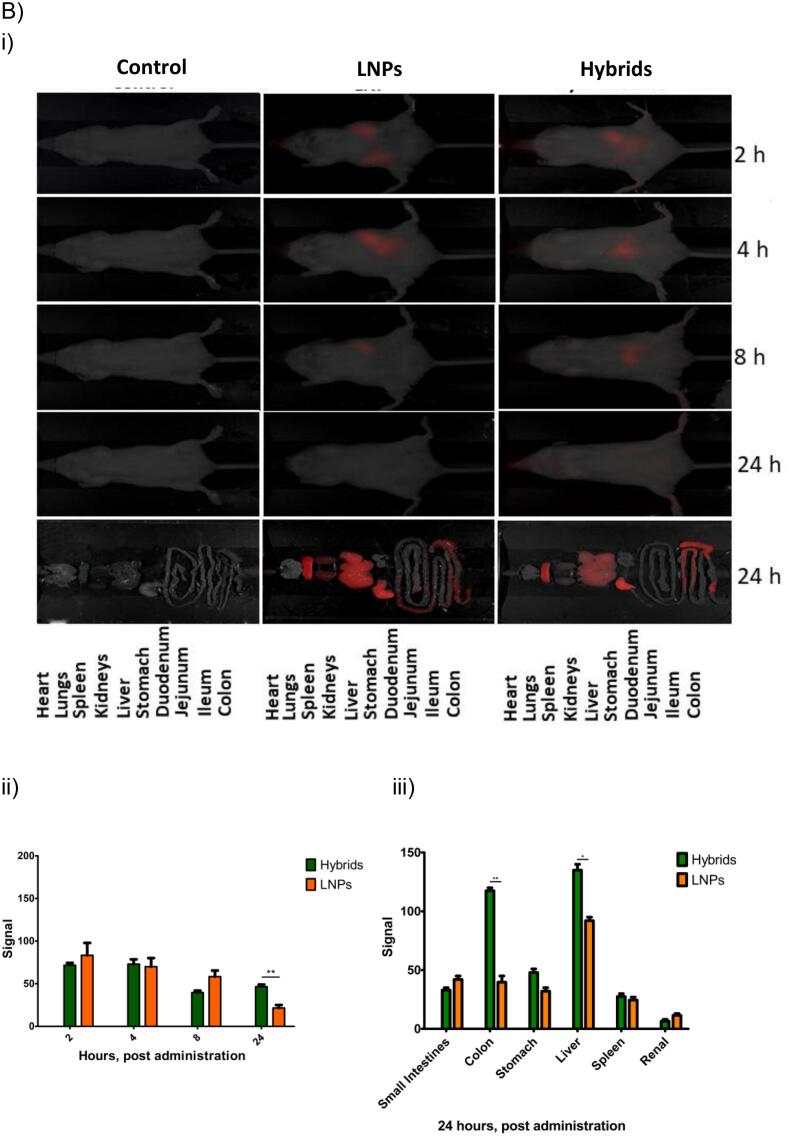
Biodistribution of orally administered mEVs, LNPs and mEV-LNP hybrids. A) BALB/c mice were administered 700 μL volume of each formulation labeled with DiR containing the same particle concentration. Administration was done through oral gavage and in vivo imaging of the mice was conducted after 2, 4, 8 and 24 h of administration using a Pearl Trilogy system. Ai) whole body imaging; Aii) fluorescence intensity at different times following oral dosing and Aiii) Quantification of fluorescence in mice organs (n = 3). B) Mice were administered 700 μL volume of each formulation labeled with IRDye® 800 CW siRNA containing the same particle concentration. Bi) whole body imaging; Bii) fluorescence intensity of IRDye® 800 CW at different times following oral dosing and Biii) Quantification of IRDye® 800 CW fluorescence in mice organs (n = 3). *p < 0.05; **p < 0.01; ***p < 0.001.

The data in Fig. 6Ai shows that, following the administration of the samples by oral gavage, DiR (green) fluorescence signal was observed in the abdomen area at 2 h imaging time point, which subsequently reduced in area and/or intensity after 4 h, for all samples. The fluorescence signal was statistically significantly stronger for mEV-LNP hybrids compared to mEVs at all the measurement intervals and higher than LNPs at 8 h (Fig. 6Aii). While this is an interesting observation, additional experiments are needed to determine the precise location of the samples, particularly whether they remain associated with the intestinal mucosa or are absorbed into the submucosal space. Considering the fluorescence signal distribution in individual organs harvested post experiment (Fig. 6Aiii), the data shows that fluorescence signal was generally apparent for all the samples in the different regions of the gastrointestinal tract, with the signal being the strongest in the stomach. Fluorescence was also observed in the liver and spleen, while a very low level was also detected in the kidneys. There was a significantly higher fluorescence intensity associated with hybrid particles compared to mEVs and LNPs in the colon, as well as higher signal intensity in the stomach for hybrid particles and LNPs compared to mEVs, while there is no difference between the samples in other organs.

The data of the parallel biodistribution study performed with fluorescently labeled siRNA (cargo), shown in Fig. 6Bi, demonstrates a similar pattern of (red) fluorescence signal in the abdomen area of the animals, which decreases in intensity and area from 2 h to 8 h measurement time points, eventually reducing substantially, attributed to concentration detection limit through the skin and tissue, at 24 h (Fig. 6Bii). In terms of organ biodistribution (Fig. 6Biii), the fluorescence signal of samples containing labeled siRNA generally showed a similar trend to the samples labeled with the lipid dye in Fig. 6A, with the fluorescence signal associated with hybrid particles in the harvested organs 24 h post administration being notably prominent and significantly higher than LNPs in the colon and liver. These observations may suggest that the mEV-LNP hybrid systems are potentially useful for RNA delivery to the colon via oral administration. This may suggest that this delivery strategy is useful for gene silencing in the colon with per oral route of administration, for example in colitis, although this needs to be confirmed in relevant animal models of the disease, given that the particles may show different distribution in the diseased tissue. At the same time, the limitations of the current study must be noted. Specifically, the use of fluorescently labeled systems and fluorescent imaging traces the fluorescent label, which may dissociate from the hybrid particles (or EVs and LNP) in the gut. However, the use of both RNA- and EV-associated fluorophores with correlated distribution, together with stability of the hybrid particles in simulated intestinal media may suggest that the hybrid particles, including the fluorescent tag, remain intact. Due to the natural origin of EVs, together with FDA-approved lipids in LNPs, it is expected that the systems are fully eliminated. Future studies should test the efficacy of the mEV-LNP hybrid systems in relevant animal disease models of both gastrointestinal and systemic diseases, particularly colitis, determining whether the systems achieve successful delivery of functional siRNA leading to a therapeutic response.

Our study is significant for several reasons. Firstly, we show that the microfluidic approach offers an efficient method that employs non-disruptive conditions for generating RNA-loaded mEV-LNP hybrids. Secondly, we report a comprehensive and deep characterization strategy (employing complementary techniques including FTIR, nFCM and super resolution microscopy) for these novel materials. Importantly, we demonstrate the delivery potential of mEV-LNP systems (gene silencing efficiency in multiple cell lines, which is retained following exposure to simulated intestinal fluids), with superior knockdown compared to LNPs. Overall, the work suggests that engineered milk extracellular vesicles may address the currently significant barrier of oral delivery of RNA therapeutics.

## 4 Conclusions

EV hybrids are a promising strategy to unlock the potential of EVs for RNA delivery. In this work we report microfluidically engineered siRNA-loaded EV-LNP hybrids, which we extensively characterized, revealing a hydrodynamic diameter close to constituent particles, a narrow PdI and high fusion efficiency. The generated EV-LNP hybrids showed an enhanced stability in digestive fluids, reduced cytotoxicity and a markedly superior oral siRNA delivery profile compared to LNPs from which they are generated. Importantly, the EV-LNP hybrid systems were shown to demonstrate enhanced accumulation in the colon compared to LNPs, which highlights their potential for oral delivery of RNA therapies for diseases affecting this gut region, such as colitis.

## Ethics approval statement

Animal studies were carried out under the University of Prishtina (Kosovo) guidelines for the care of experimental animals and were approved by Research and Ethics Committee of the Faculty of Medicine.

## Funding statement

This work was funded by the King's-China Scholarship Council PhD Scholarship Programme, the European Union (EuropeAid, grant no. EUROPEAID/173691/DD/ACT/XK) and BBSRC Engineering Biology Mission Award (BB/Y008065/1). The Aston Institute for Membrane Excellence (AIME) is funded by UKRI’s Research England as part of their Expanding Excellence in England (E3) fund.

## CRediT authorship contribution statement

**Ning Ding:** Writing – original draft, Investigation, Funding acquisition. **Armond Daci:** Writing – original draft, Investigation, Funding acquisition. **Vanesa Krasniqi:** Investigation. **Rachel Butler:** Investigation. **Alan Goddard:** Supervision, Funding acquisition. **Qing Guo:** Investigation. **Yunyue Zhang:** Investigation. **Jizhou Zhong:** Investigation. **K.L. Andrew Chan:** Writing – original draft, Supervision. **Maya Thanou:** Writing – original draft, Supervision. **Driton Vllasaliu:** Writing – review & editing, Writing – original draft, Visualization, Supervision, Funding acquisition, Conceptualization.

## Declaration of competing interest

The authors declare no conflict of interest.

## Data Availability

The data that support the findings of this study are available from the corresponding author upon reasonable request.

## Appendix A Supplementary data

Supplementary material Image 1

## References

[bb0005] Aslan C., Kiaie S.H., Zolbanin N.M., Lotfinejad P., Ramezani R., Kashanchi F., Jafari R. (2021). Exosomes for mRNA delivery: a novel biotherapeutic strategy with hurdles and hope. BMC Biotechnol..

[bb0010] Ball R.L., Bajaj P., Whitehead K.A. (2018). Oral delivery of siRNA lipid nanoparticles: Fate in the GI tract. Sci. Rep..

[bb0015] Bannunah A., Cavanagh R., Shubber S., Vllasaliu D., Stolnik S. (2024). Difference in endocytosis pathways used by differentiated versus nondifferentiated epithelial Caco-2 cells to internalize nanosized particles. Mol. Pharm..

[bb0020] Bohsen M.S., Tychsen S.T., Kadhim A.A.H., Grohganz H., Treusch A.H., Brandl M. (2023). Interaction of liposomes with bile salts investigated by asymmetric flow field-flow fractionation (AF4): a novel approach for stability assessment of oral drug carriers. Eur. J. Pharm. Sci..

[bb0025] Clayburgh, D.R., Turner, J.R., 2005.

[bb0030] de Jong O.G., Kooijmans S.A.A., Murphy D.E., Jiang L., Evers M.J.W., Sluijter J.P.G., Vader P., Schiffelers R.M. (2019). Drug delivery with extracellular vesicles: from imagination to innovation. Acc. Chem. Res..

[bb0035] Ducrot C., Loiseau S., Wong C., Madec E., Volatron J., Piffoux M. (2023). Hybrid extracellular vesicles for drug delivery. Cancer Lett..

[bb0040] Fu S., Wang Y., Xia X., Zheng J.C. (2020). Exosome engineering: current progress in cargo loading and targeted delivery. NanoImpact.

[bb0045] Gandek T.B., van der Koog L., Nagelkerke A. (2023). A comparison of cellular uptake mechanisms, delivery efficacy, and intracellular fate between liposomes and extracellular vesicles. Adv. Healthc. Mater..

[bb0050] Hald Albertsen C., Kulkarni J.A., Witzigmann D., Lind M., Petersson K., Simonsen J.B. (2022). The role of lipid components in lipid nanoparticles for vaccines and gene therapy. Adv. Drug Deliv. Rev..

[bb0055] Herrmann I.K., Wood M.J.A., Fuhrmann G. (2021). Extracellular vesicles as a next-generation drug delivery platform. Nat. Nanotechnol..

[bb0060] Hou X., Zaks T., Langer R., Dong Y. (2021). Lipid nanoparticles for mRNA delivery. Nat. Rev. Mater..

[bb0065] Jash A., Ubeyitogullari A., Rizvi S.S.H. (2021). Liposomes for oral delivery of protein and peptide-based therapeutics: challenges, formulation strategies, and advances. J. Mater. Chem. B.

[bb0070] Kim H., Kim D.E., Han G., Lim N.R., Kim E.H., Jang Y., Cho H., Jang H., Kim K.H., Kim S.H., Yang Y. (2022). Harnessing the natural healing power of colostrum: bovine milk-derived extracellular vesicles from colostrum facilitating the transition from inflammation to tissue regeneration for accelerating cutaneous wound healing. Adv. Healthc. Mater..

[bb0075] Kupsco A., Prada D., Valvi D., Hu L., Petersen M.S., Coull B., Grandjean P., Weihe P., Baccarelli A.A. (2021). Human milk extracellular vesicle miRNA expression and associations with maternal characteristics in a population-based cohort from the Faroe Islands. Sci. Rep..

[bb0080] Lee Y., Jeong M., Park J., Jung H., Lee H. (2023). Immunogenicity of lipid nanoparticles and its impact on the efficacy of mRNA vaccines and therapeutics. Exp. Mol. Med..

[bb0085] Luo X., Zhang Y., Ding N., Javorovic J., Raimi-Abraham B.T., Lynham S., Yang X., Shenker N., Vllasaliu D. (2025). Mechanistic insight into human milk extracellular vesicle-intestinal barrier interactions. J. Extracell Biol..

[bb0090] Mihaly J., Deak R., Szigyarto I.C., Bota A., Beke-Somfai T., Varga Z. (2017). Characterization of extracellular vesicles by IR spectroscopy: Fast and simple classification based on amide and CH stretching vibrations. Biochim. Biophys. Acta Biomembr..

[bb0095] Moghimi S.M., Simberg D. (2022). Pro-inflammatory concerns with lipid nanoparticles. Mol. Ther..

[bb0100] Murphy D.E., de Jong O.G., Evers M.J.W., Nurazizah M., Schiffelers R.M., Vader P. (2021). Natural or synthetic RNA delivery: a stoichiometric comparison of extracellular vesicles and synthetic nanoparticles. Nano Lett..

[bb0105] Parlati F., Weber T., McNew J.A., Westermann B., Sollner T.H., Rothman J.E. (1999). Rapid and efficient fusion of phospholipid vesicles by the alpha-helical core of a SNARE complex in the absence of an N-terminal regulatory domain. Proc. Natl. Acad. Sci. USA.

[bb0110] Rodriguez D.A., Vader P. (2022). Extracellular vesicle-based hybrid systems for advanced drug delivery. Pharmaceutics.

[bb0115] Samuel M., Fonseka P., Sanwlani R., Gangoda L., Chee S.H., Keerthikumar S., Spurling A., Chitti S.V., Zanker D., Ang C.S., Atukorala I., Kang T., Shahi S., Marzan A.L., Nedeva C., Vennin C., Lucas M.C., Cheng L., Herrmann D., Pathan M., Chisanga D., Warren S.C., Zhao K., Abraham N., Anand S., Boukouris S., Adda C.G., Jiang L., Shekhar T.M., Baschuk N., Hawkins C.J., Johnston A.J., Orian J.M., Hoogenraad N.J., Poon I.K., Hill A.F., Jois M., Timpson P., Parker B.S., Mathivanan S. (2021). Oral administration of bovine milk-derived extracellular vesicles induces senescence in the primary tumor but accelerates cancer metastasis. Nat. Commun..

[bb0120] Sato Y.T., Umezaki K., Sawada S., Mukai S.A., Sasaki Y., Harada N., Shiku H., Akiyoshi K. (2016). Engineering hybrid exosomes by membrane fusion with liposomes. Sci. Rep..

[bb0125] Sheridan C. (2023). Why gene therapies must go virus-free. Nat. Biotechnol..

[bb0130] Sutaria D.S., Jiang J., Elgamal O.A., Pomeroy S.M., Badawi M., Zhu X., Pavlovicz R., Azevedo-Pouly A.C.P., Chalmers J., Li C., Phelps M.A., Schmittgen T.D. (2017). Low active loading of cargo into engineered extracellular vesicles results in inefficient miRNA mimic delivery. J. Extracell Vesicles.

[bb0135] van Herwijnen M.J., Zonneveld M.I., Goerdayal S., Nolte-’t Hoen E.N., Garssen J., Stahl B., Maarten Altelaar A.F., Redegeld F.A., Wauben M.H. (2016). Comprehensive proteomic analysis of human milk-derived extracellular vesicles unveils a novel functional proteome distinct from other milk components. Mol. Cell. Proteomics.

[bb0140] Zhang C., Ma Y., Zhang J., Kuo J.C., Zhang Z., Xie H., Zhu J., Liu T. (2022). Modification of lipid-based nanoparticles: an efficient delivery system for nucleic acid-based immunotherapy. Molecules.

[bb0145] Zhang Y., Belaid M., Luo X., Daci A., Limani R., Mantaj J., Zilbauer M., Nayak K., Vllasaliu D. (2023). Probing milk extracellular vesicles for intestinal delivery of RNA therapies. J. Nanobiotechnol..

[bb0150] Zhang Y., Luo X., Ding N., Belaid M., Thanou M., Vllasaliu D. (2024). Hybrid milk extracellular vesicles as potential systems for oral delivery of siRNA. Advan. Therapeut..

