# Supplementary material for: Engineered extracellular vesicles demonstrate altered endocytosis and biodistribution and have superior oral siRNA delivery efficiency compared to lipid nanoparticles
Source: Int J Pharm X. 2025 Oct 26;10:100428. doi: 10.1016/j.ijpx.2025.100428 (PMC12621563; doi:10.1016/j.ijpx.2025.100428)
Supplement: Supplementary file 1 — Supplementary material [file mmc1.docx]

**Engineered Extracellular Vesicles Demonstrate Altered Endocytosis and Biodistribution and Have Superior Oral siRNA Delivery Efficiency Compared to Lipid Nanoparticles**

Supporting Information


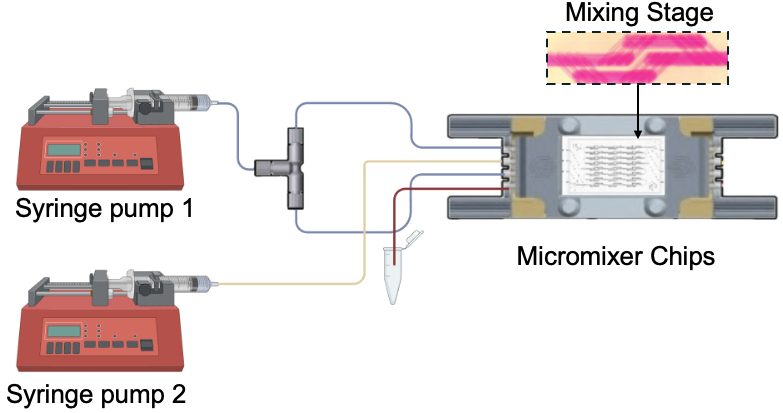


**Figure S1.** *Schematic representation of the microfluidic system used for hybrid nanoparticle preparation. Two syringe pumps (Syringe pump 1 and Syringe pump 2) deliver aqueous and organic phases into a T-junction connector, followed by introduction into the micromixer chip (Hybrid S). Within the micromixer channel, rapid mixing occurs at the designated mixing stage (pink color), leading to the formation of hybrid particles. The schematic illustrates key components including syringe pumps, connectors, micromixer chip, and the mixing stage.*

***Table S1.*** *Hydrodynamic diameter* *(nm), yield (particles/mL) and protein concentration (µg/mL) of mEVs, as measured using nanoparticle tracking analysis instrument (data expressed as mean +/- SD; n=3). Protein concentration measured using the Bicinchoninic acid (BCA) assay.*

| EV Type | Hydrodynamic diameter (nm) | Yield (Particle/mL) | Protein (mg/mL) | Zeta Potential (mV) |
| --- | --- | --- | --- | --- |
| Skimmed cow milk | 154±25.9 | 4.91×10^15^±1.14 ×10^14^ | 5.15±0.017 | -9.668±0.871 |

A) B)


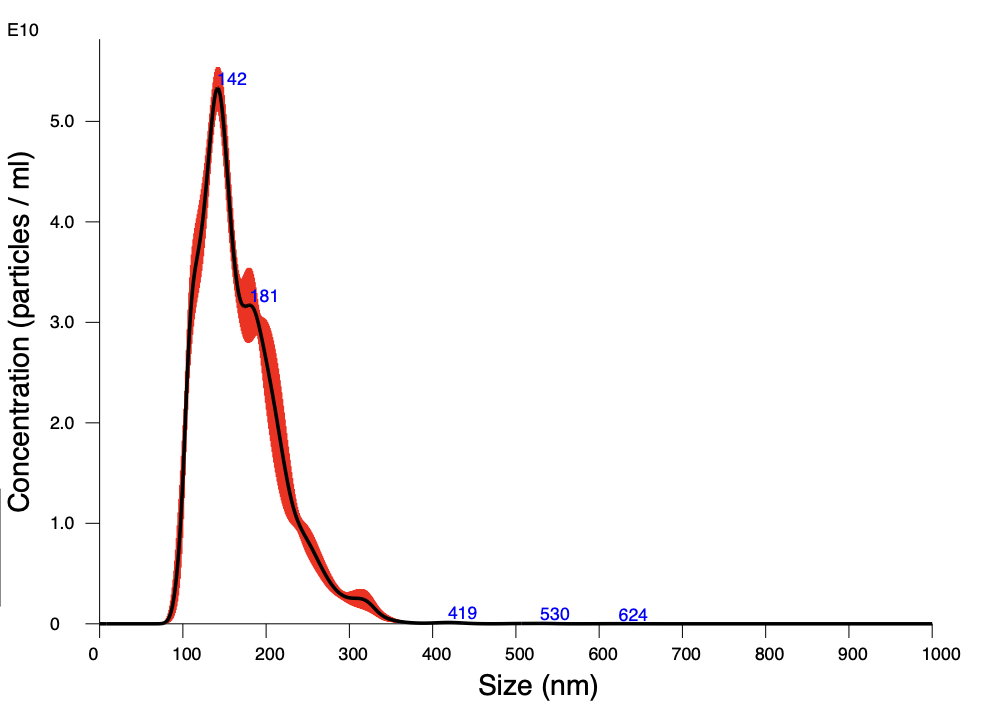

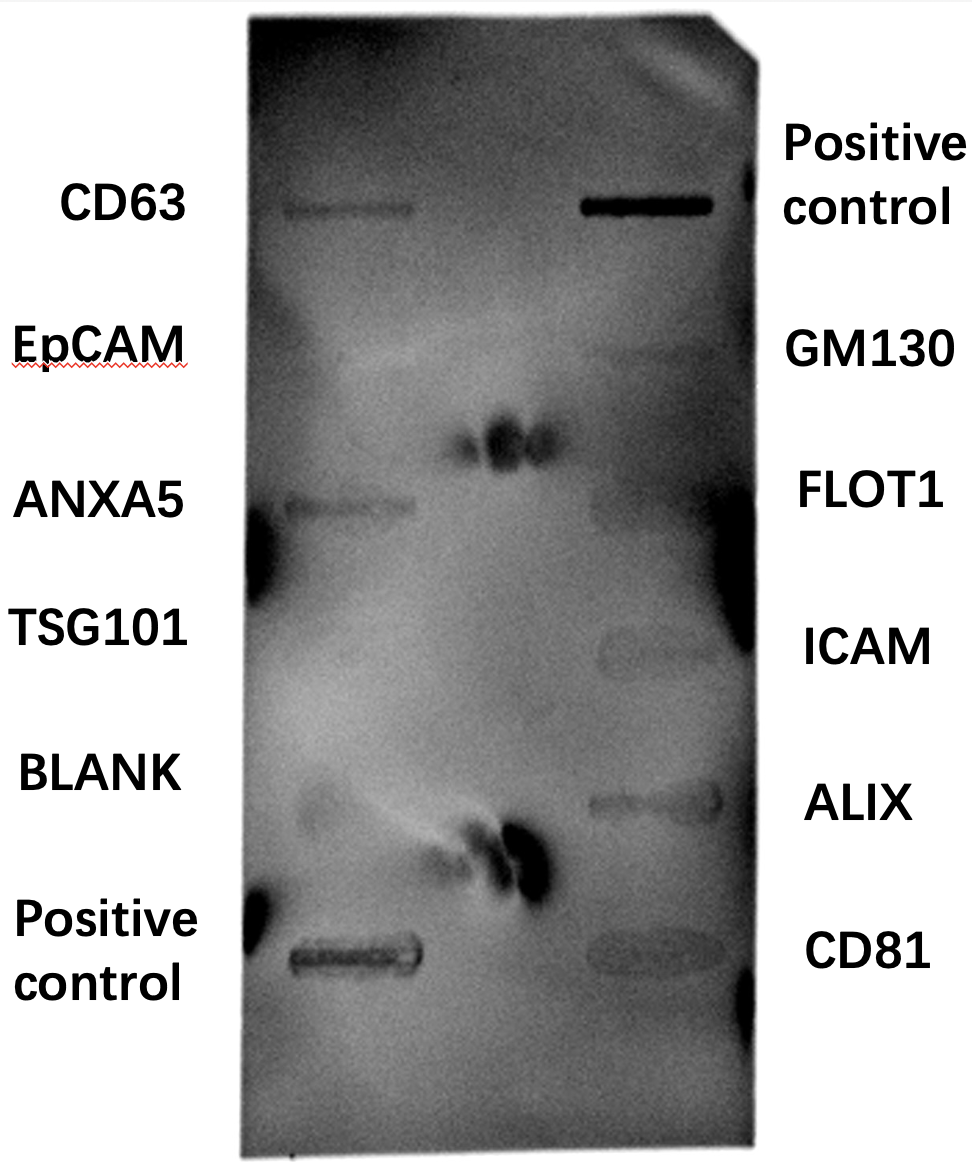


C)


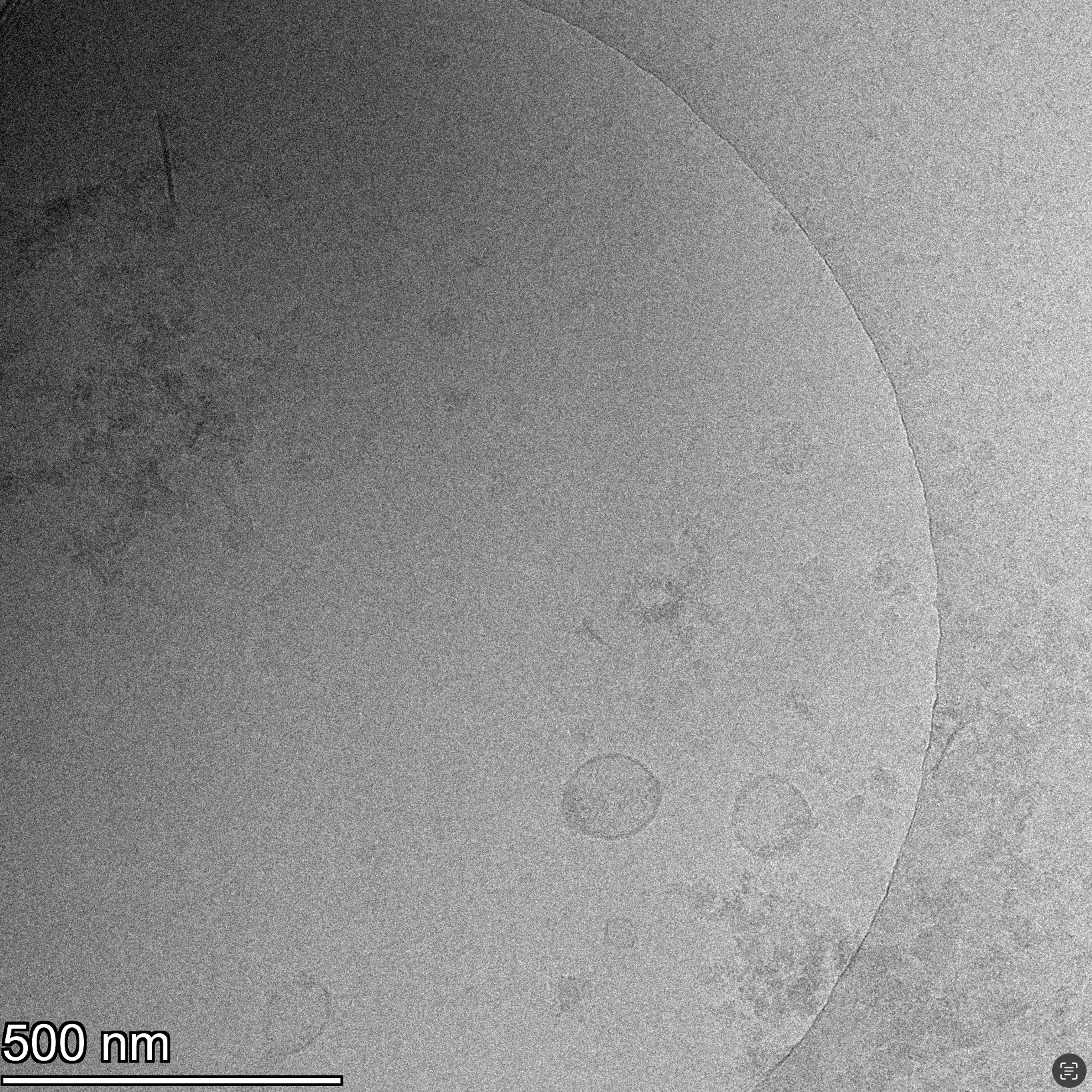


**Figure S2.** **Characterisation of EVs.** A) Nanoparticle tracking analysis (NTA) graph of mEVs isolated by ultracentrifugation. mEVs were diluted 1000 times with filtered PBS. Data are shown as mean +/- SD (n=3). B) Expression of specific markers analysis on bovine milk extracellular vesicles isolated via ultracentrifugation using the Exo-check Exosomes antibody Array^TM^. The 'positive' label indicates a successful horseradish peroxidase (HRP) detection, validating the functionality of detection reagents. The 'blank' line serves as a background control. The presence of dark bands signifies the detection of specific marker proteins including the FLOT1 (*(Flotillin 1), ICAM (Intercellular Adhesion Molecule-1), CD81, CD63, ANX45 (Annexin V) C) Cryogenic transmission electron microscopy (Cryo-TEM) image of milk-derived extracellular vesicles (mEVs) isolated by differential ultracentrifugation.*

### Preparation and Characterization of siRNA-loaded LNPs

***Table S2. Characterisation of LNPs.*** siRNA-loaded particles *hydrodynamic diameter* and surface charge characteristics were measured using a Zetasizer (Malvern Instruments). siRNA entrapment efficiency was assessed using fluorescence-labelled siRNA. Surface charge measurements were conducted in 20 mM sodium acetate buffer at pH 4.

|  | Molar Ratio (%) | Mean hydrodynamic diameter (d.nm) | PDI | Zeta Potential (mV) | EE (%) |
| --- | --- | --- | --- | --- | --- |
| SM-102/DSPC/Cholesterol/PEG-PE | 50/10/38.5/1.5 | 163.5 ± 6.7 | 0.101 ± 0.059 | 46.5 ± 1.9 | 90 |
| ALC-0315/DSPC/ Cholesterol/PEG-PE | 50/10/38.5/1.5 | 161.5 ± 3.1 | 0.226 ± 0.021 | 33.5 ± 4.3 | 85 |


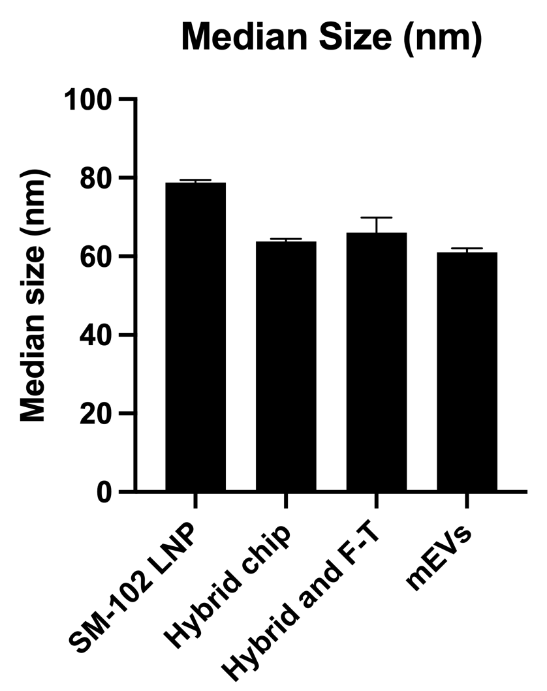


**Figure S3. Median particle size (nm) of SM-102 lipid nanoparticles, hybrid nanoparticles, and milk-derived EVs prepared using different methods.** Methods include microfluidic chip fabrication and conventional freeze–thaw (F–T) cycles. Size measurements were performed using NanoFCM. Mean± SD.

A)

**

B)


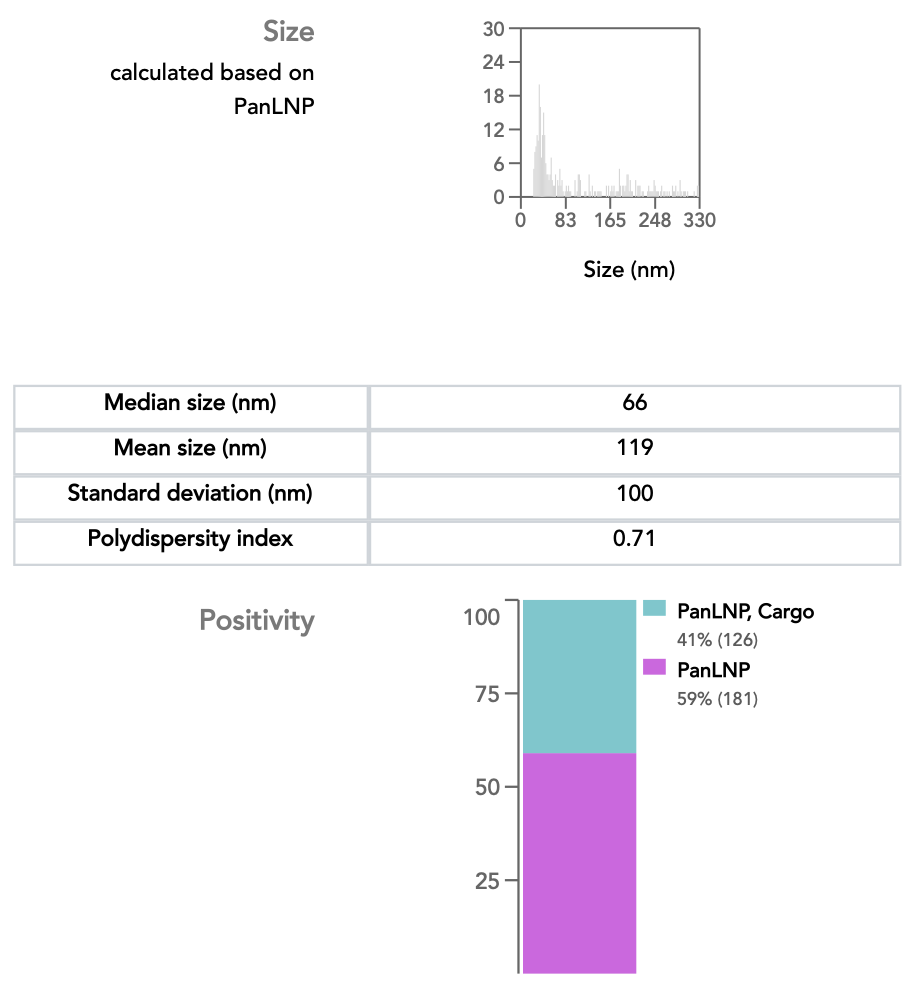


**Figure S4. Qualitative and quantitative characterization of LNPs using super resolution imaging and the ONI CODI analysis platform. A)** *Super-resolution image of hybrid nanoparticles composed of mEVs and LNPs co-loaded with siRNA. LNPs are labeled with a far-red dye (647 nm), siRNA is visualized using a green cargo dye (488 nm), and mEVs are labeled with Aco-600™ (yellow). The composite spatial distribution highlights colocalization of all components within the hybrid structure. Scale bar: 400 nm.* Imaging performed with an ONI NanoImager. **B)** *Lipid nanoparticles (LNPs) encapsulating fluorescently labeled siRNA were characterized using the CODI analysis platform developed by ONI (https://alto.codi.bio), which provides single-particle quantification of nanoparticle integrity, size, and fluorescent marker co-localization using advanced fluorescence microscopy and automated particle classification.*

**
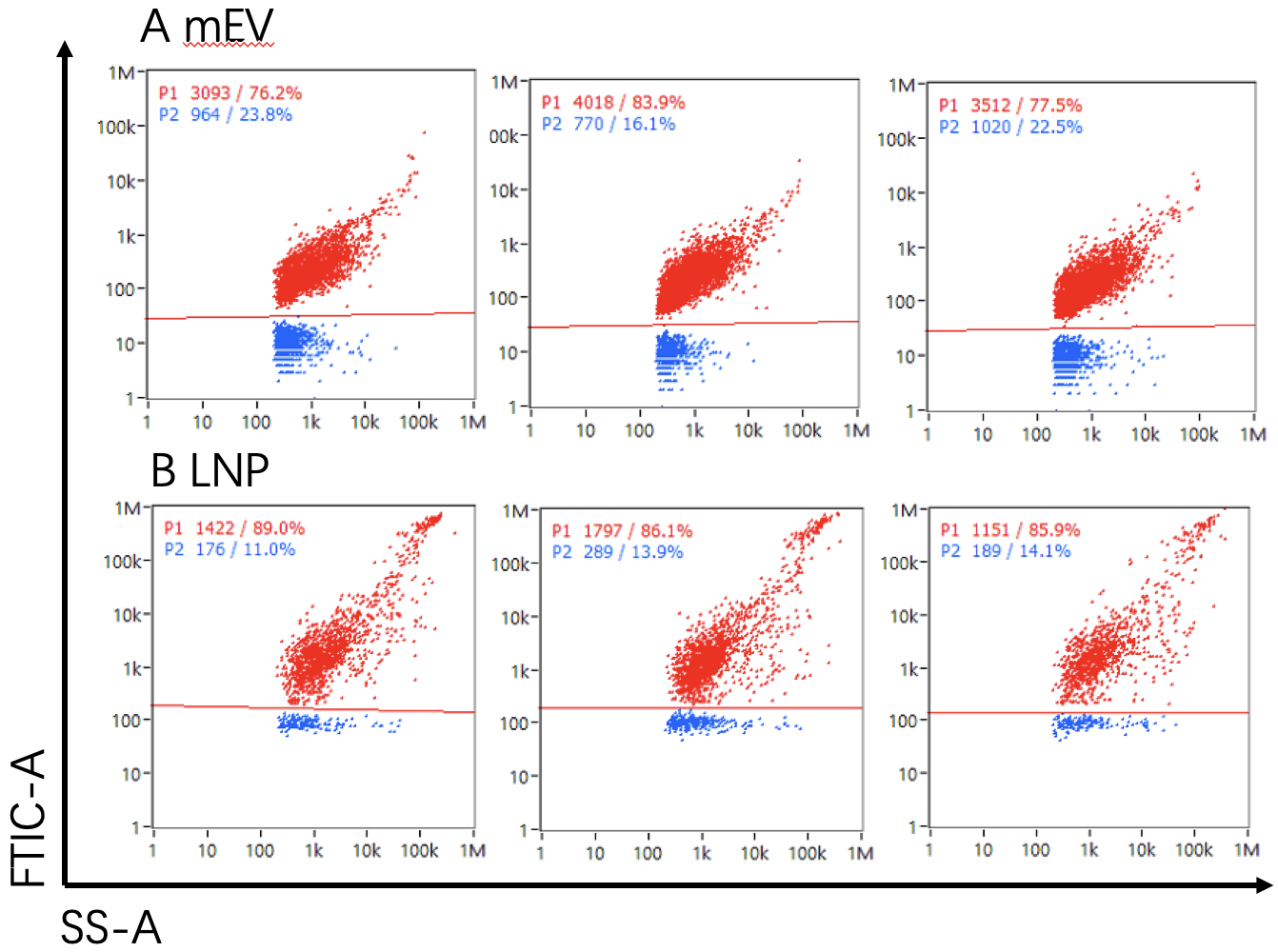
**

**
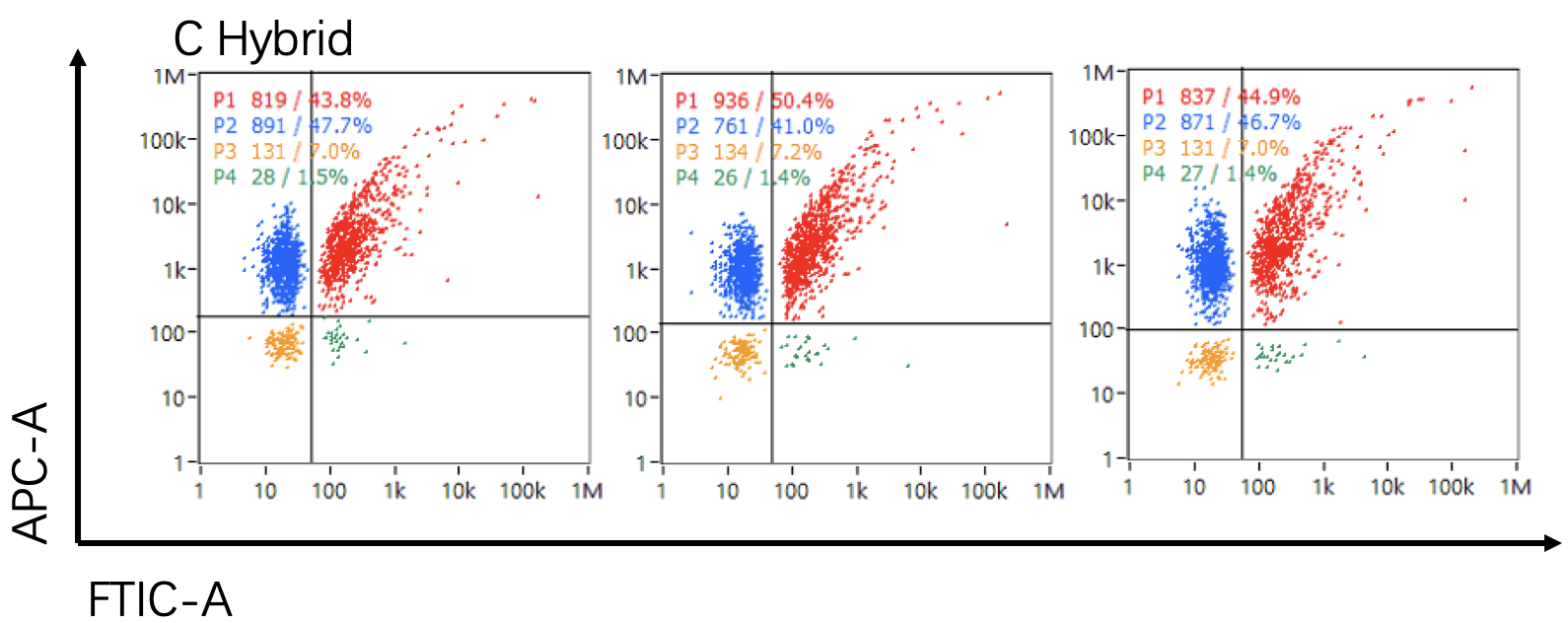
**

**Figure S5. Representative dot plots of LNPs, mEVs and Hybrids.** The experiments were carried out in triplicate.

| Nuclei | Hybrid | Overlay |
| --- | --- | --- |
| 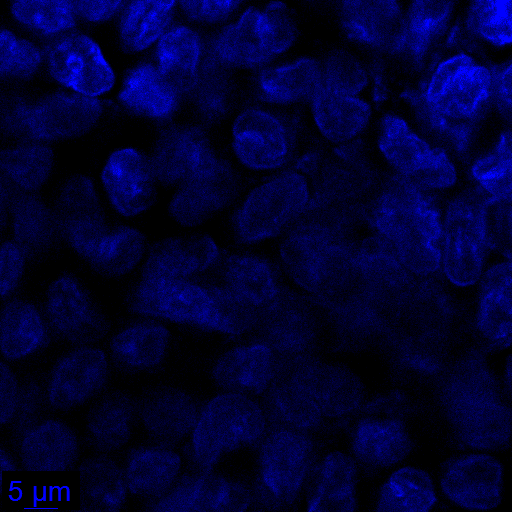 | 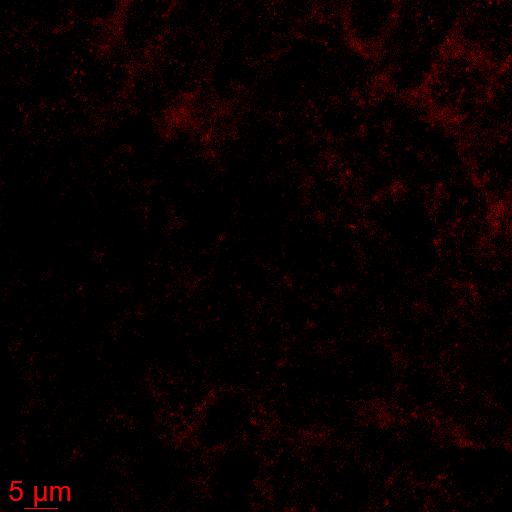 | 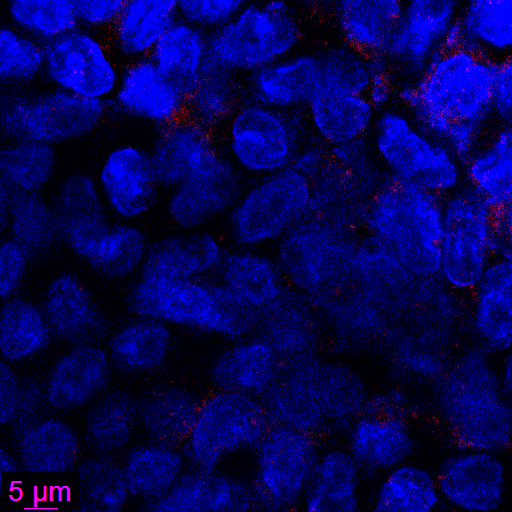 |

**Figure S6. Uptake of labelled mEV-LNP hybrids in differentiated Caco-2 monolayers***.Nanoparticles were diluted in HEPES buffer to a final concentration of 0.1 mg/mL. Cells were incubated with samples for 3 hours. Fluorescence images were acquired using a Leica Confocal Microscope. Blue: nuclei stain (DAPI); Red: Hybrid labelled with Aco-600^TM^. Scale bar, 5µm.*

**Figure S7. Effect of formulations on intestinal epithelial transport of siRNA.** *Transport of fluorescent Cy5 siRNA loaded in mEVs by electroporation (‘mEV(ele)’), LNP-mEV hybrids (based on SM102 lipid) generated by microfluidic mixing (‘hybrid chip’) and freeze-thawing (‘hybrid F-T’), and siRNA alone in differentiated (polarized) intestinal Caco-2 monolayers. Transport percentage was calculated by the fluorescent signal of Cy5-siRNA. Data shown as the mean ± SD (n=3). Transwell permeable cell insert (polycarbonate filter, 1.12 cm^2^ area, 12 mm diameter, 0.4 μm pore size). *p < 0.05*

***Table S3. Effect of treatment with fed- and fasted-state simulated intestinal fluid (FaSSIF and FeSSIF, respectively) on particle hydrodynamic diameter.*** *Samples were incubated with FaSSIF or FeSSIF for 1.5 h, followed by recovery via ultracentrifugation. Hydrodynamic diameter determined by dynamic light scattering. Data shown as the mean ± SD (n=3). Statistical analysis (paired t-test comparison between particles before and after treatment.* *p < 0.05; **p < 0.01.

| **Sample / Treatment** | **Z-Average (d.nm) before treatment** | **PDI** | **Z-Average (d.nm) after treatment** | **PDI** |
| --- | --- | --- | --- | --- |
| mEV / FaSSIF | **82.8±0.87** | **0.393±0.002** | **139.4±5**** | **0.409 ± 0.079** |
| mEV / FeSSIF | **102±0.56** | **0.295±0.04** | **224.0±10**** | **0.734 ± 0.040** |
| Hybrid / FaSSIF | **160.97 ± 2.87** | **0.154 ± 0.005** | **177.4±7*** | **0.785 ± 0.054** |
| Hybrid / FeSSIF | **160.9±7.52** | **0.255±0.007** | **320.0±35**** | **0.781 ± 0.200** |

A)

B)

|  | Hybrid A | Hybrid S | Positive control | eGFP siRNA only |
| --- | --- | --- | --- | --- |
| t0h | 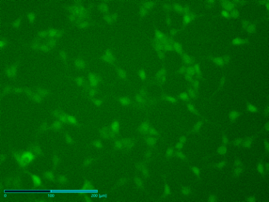 | 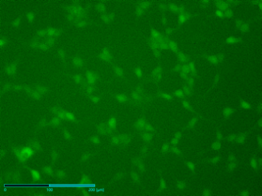 | 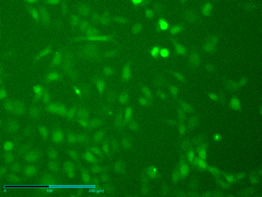 | 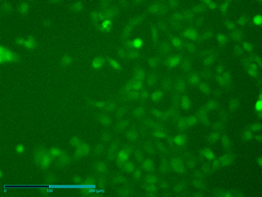 |
|  | 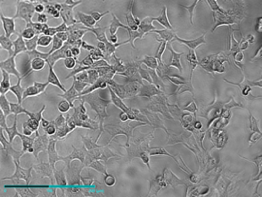 | 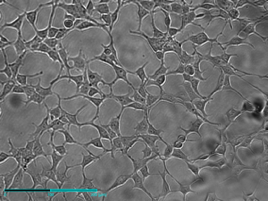 | 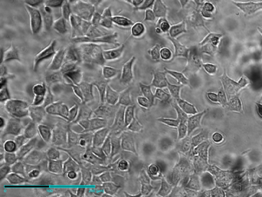 | 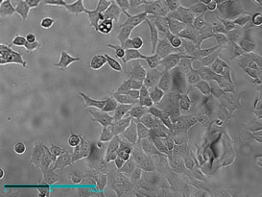 |
| t42h | 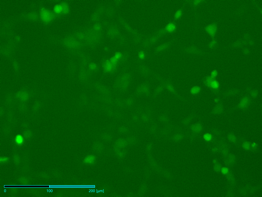 | 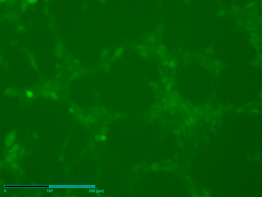 | 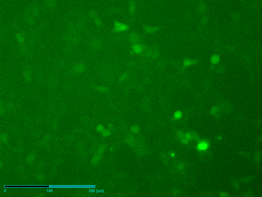 | 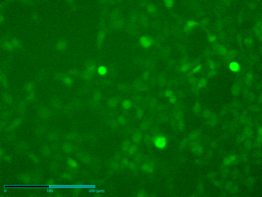 |
|  | 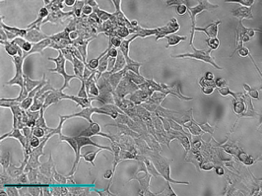 | 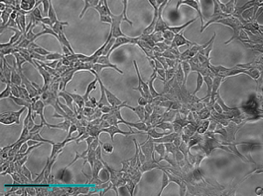 | 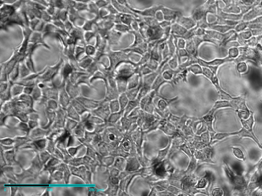 | 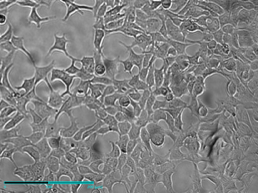 |

**Figure S8**. **In vitro GFP silencing efficiency in HEK293-GFP cells. A)** *Cells were treated with GFP siRNA delivered by microfluidic LNP–mEV hybrids formulated with ALC-0315 (‘Hybrid A’), SM-102 (‘Hybrid S’), or with siRNA alone. Lipofectamine CRISPRMAXTM Transfection Reagent was used as a positive control with siRNA concentration of 50 nM. Intervention was applied for 42 h, and images were acquired every 8 h using the AutoLCI live-cell imaging system. Mean fluorescence intensity was obtained by ImageJ software****.****Bars show mean ± SD from n = 3 independent experiments (each including technical replicates). Statistics carried out using two-way ANOVA;** indicates p < 0.05, **** indicates p < 0.0001 and ‘ns’= non-significant. **B)** Representative fluorescence image of HEK 293 cells before and after 42 hours transfection (‘before intervention’ and ‘after intervention’, respectively) with siRNA-loaded hybrids.
